# Psychological therapy outcomes by sexual orientation and gender: a retrospective cohort study

**DOI:** 10.1017/S0033291725101220

**Published:** 2025-07-24

**Authors:** Tom Kent, Jae W. Suh, Glyn Lewis, Rob Saunders, Neil M. Davies, Gemma Lewis, Lauren Dolby, Joshua E. J. Buckman

**Affiliations:** 1 Division of Psychiatry, University College London, London, UK; 2 Department of Psychology, Royal Holloway, University of London, London, UK; 3 CORE Data Lab, Centre for Outcomes Research and Effectiveness (CORE), Research Department of Clinical, Educational & Health Psychology, University College London, London, UK; 4 Department of Statistical Science, University College London, London, UK; 5 Department of Public and Nursing, Norwegian University of Science and Technology – NTNU, Trondheim, Norway; 6 NHS Talking Therapies for Anxiety and Depression Services, North London NHS Foundation Trust, St Pancras Hospital, London, UK

**Keywords:** anxiety, depression, psychological therapies, sexual minorities, sexual orientation

## Abstract

**Background:**

Lesbian, gay, and bisexual (LGB) individuals are more than twice as likely to experience anxiety and depression compared with heterosexuals. Minority stress theory posits that stigma and discrimination contribute to chronic stress, potentially affecting clinical treatment. We compared psychological therapy outcomes between LGB and heterosexual patients by gender.

**Methods:**

Retrospective cohort data were obtained from seven NHS talking therapy services in London, from April 2013 to December 2023. Of 100,389 patients, 94,239 reported sexual orientation, 7,422 identifying as LGB. The primary outcome was reliable recovery from anxiety and depression. Secondary outcomes were reliable improvement, depression and anxiety severity, therapy attrition, and engagement. Analyses were stratified by gender and employed multilevel regression models, adjusting for sociodemographic and clinical covariates.

**Results:**

After adjustment, gay men had higher odds of reliable recovery (OR: 1.23, 95% CI: 1.13–1.34) and reliable improvement (OR: 1.16, 95% CI: 1.06–1.28) than heterosexual men, with lower attrition (OR: 0.88, 95% CI: 0.80–0.97) and greater reductions in depression (MD: 0.51, 95% CI: 0.28–0.74) and anxiety (MD: 0.45, 95% CI: 0.25–0.65). Bisexual men (OR: 0.67, 95% CI: 0.54–0.83) and bisexual women (OR: 0.84, 95% CI: 0.77–0.93) had lower attrition than heterosexuals. Lesbian and bisexual women, and bisexual men, attended slightly more sessions (MD: 0.02–0.03, 95% CI: 0.01–0.04) than heterosexual patients. No other differences were observed.

**Conclusions:**

Despite significant mental health burdens and stressors, LGB individuals had similar, if not marginally better, outcomes and engagement with psychological therapy compared with heterosexual patients.

## Introduction

Lesbian, gay, and bisexual (LGB) individuals, also referred to as sexual minorities, experience about double the rate of common mental disorders (CMDs), such as depression and anxiety, compared with heterosexuals (Amos et al., 2020; King et al., 2008; Pitman et al., 2022; Plöderl & Tremblay, 2015). Minority stress theory hypothesizes that this increased risk is a result of chronic stress caused by stigma, prejudice, and discrimination, which heighten vulnerability to mental health issues (Brooks, 1981; Meyer, 2003).

Psychological therapies are a front-line treatment for CMDs (Clark, 2011) and have demonstrated effectiveness compared to control conditions (Cuijpers et al., 2024), offering significant benefits for many patients. However, minority stress processes may undermine therapeutic outcomes and engagement for LGB individuals. Chronic exposure to stigma-related stressors can heighten maladaptive psychological processes, including rumination and emotion dysregulation (Hatzenbuehler, 2009), both of which have been associated with poorer psychotherapy outcomes (Bar-Sella et al., 2023; Jones, Siegle, & Thase, 2008). In addition, entrenched expectations of rejection (Feinstein, 2020), the need to conceal their identity in unsupportive environments (Maji, Yadav, & Gupta, 2023), and discrimination within healthcare settings (Ayhan et al., 2020; Meads, Hunt, Martin, & Varney, 2019) may reduce trust in clinicians and inhibit openness. Together, these factors may contribute to differences in treatment response and engagement between LGB and heterosexual patients.

In England, psychological therapies are primarily delivered through the National Health Service’s Talking Therapies for Anxiety and Depression Services (TTads; formerly Improving Access to Psychological Therapies), the country’s largest provider of evidence-based treatments for common mental disorders. TTads delivers millions of therapy sessions annually and forms a core component of publicly funded mental health care (Clark, 2018; NHS Digital, 2023). In 2023, more than 1.5 million people used these services, with around 700,000 completing a course of therapy (NHS Digital, 2023).

However, previous studies found differences in treatment outcomes for some sexual minority groups within TTads. A South London study found evidence that bisexual women were less likely to meet the criteria for reliable recovery (a TTads metric based on threshold criteria for improvement in depression and anxiety) after psychological therapy compared to heterosexual women. In contrast, there was little evidence of a difference between lesbian and heterosexual women or between gay and heterosexual men (Rimes et al., 2018). A subsequent study, which examined a national dataset from TTads, found evidence that bisexual men and women were less likely to meet reliable recovery criteria after psychological therapy compared to their heterosexual counterparts, and had higher final-session severity scores. Lesbian women also had lower reliable recovery and similarly elevated final-session scores. However, there was little evidence of a difference between gay and heterosexual men (Rimes, Ion, Wingrove, & Carter, 2019).

The findings, based on data collected from 2012 to 2015, may no longer capture current clinical practices or societal contexts. During that period, data on sexual orientation were inconsistently recorded, with some individuals not asked about their orientation. In the previously referenced study (Rimes et al., 2019), 33% of sexual orientation data were missing. Routine data collection has since improved, as the systematic recording of sexual orientation now allows for more precise estimates. Additionally, previous research focused on recovery rates, overlooking other important outcomes such as therapy engagement and attrition rates, which are critical for understanding treatment disparities.

While UK-based findings suggest disparities in therapy outcomes for some sexual minority groups, international studies conducted in more intensive clinical settings report a different pattern. In Europe, research from inpatient and crisis intervention services has found no differences in outcomes between LGB and heterosexual patients (Plöderl et al., 2017; Plöderl, Mestel, & Fartacek, 2022). Similarly, studies from the United States, including those in day hospitals for eating disorders and residential programs for obsessive-compulsive and related disorders, have generally shown comparable outcomes across sexual orientation groups (Bezahler et al., 2022; Donahue et al., 2020). Nonetheless, some disparities have been documented; for example, bisexual individuals have reported higher rates of self-injurious thoughts and poorer perceived quality of care following partial hospitalization (Beard et al., 2017). These international studies, however, were typically conducted in highly structured, resource-intensive environments involving multiple clinicians and adjunctive interventions. They were also often limited by small sample sizes and the aggregation of diverse sexual and gender minority groups, potentially obscuring subgroup-specific differences. As such, their findings may not be generalizable to routine psychological therapy services in the UK.

Beyond the patterns observed in prior evidence, broader social and clinical changes may be shaping therapy outcomes for LGB individuals. Over the past decade, societal acceptance of same-sex relationships has increased (Huchet-Bodet, Albakri, & Smith, 2019), and more individuals now openly identify as LGB in national surveys (Office for National Statistics, 2022). The introduction of the 2013 Marriage (Same-Sex Couples) Act in England and Wales marked an important equity milestone and has been associated with better mental health functioning for LGB individuals in later years (Teo, Metheny, & Chum, 2022). Additionally, new guidelines have been developed to improve culturally competent care for LGB clients (Barker, 2019; Beattie & Laville, 2024; Richards, Gibson, Jamieson, & Lenihan, 2019). These factors could influence therapy outcomes for LGB individuals.

Considering these changes, using contemporary data is essential for determining whether disparities persist in CMD treatment outcomes among LGB individuals. Data from seven TTads were used to (1) describe pretreatment characteristics and treatment outcomes for depression and anxiety between LGB and heterosexual patients, stratified by gender and (2) evaluate differences in recovery, improvement, engagement, and attrition rates between these groups.

### Materials and methods

We preregistered a protocol and analysis plan (https://osf.io/ydgsq/). Post hoc deviations were made to improve rigor. Hypothesis-free baseline tests (e.g. ANOVA, chi-square) were omitted due to limited interpretative validity. Treatment-related variables (e.g. session frequency, intensity) and cohort-specific factors were excluded from the main analysis to prevent overadjustment bias, as they may act as mediators. Instead, we report these analyses in the Supplementary Materials (Tables 5 and 6). Participants with missing gender data were excluded, as this variable was needed for stratification. The analysis code is available at https://github.com/jae-suh74/ttad-outcomes-lgb/.

### Dataset and services

We used routinely collected data from seven NHS TTads within the North and Central East London TTads Service Improvement and Research Network (Saunders et al., 2020). The data include clinical outcome measures for each patient and sociodemographic information, including sexual orientation. Each service provides treatment across one or two London boroughs and was included in the analysis due to its participation in this established research network, which had data-sharing agreements permitting service evaluation.

These services provide psychological therapies for individuals with depression and anxiety disorders, following the guidelines of the National Institute for Health and Care Excellence (Clark, 2018). The stepped care model offers two levels of intensity. “Low-intensity” therapies involve guided self-help, typically 4–8 sessions. For patients who do not respond to initial treatments or for whom such treatments are unsuitable, “high-intensity” therapies are available, usually comprising 10–20 sessions. These high-intensity therapies include clinician-led cognitive behavioral therapy (CBT), interpersonal psychotherapy, counseling for depression, and dynamic interpersonal therapy. Additional details on the service model can be found in Clark (2018).

In TTads, problem descriptors based on ICD-10 diagnostic criteria (World Health Organization, 1993) are used to identify the primary problem for treatment. The descriptor may not represent the most severe or only diagnosis; it is the agreed-upon focus of treatment. Treatment decisions are made collaboratively between the patient and the clinician, and only those treatments recommended in clinical guidelines are offered (Clark, 2018).

### Participants

We created a retrospective cohort of patients from seven North and Central East London NHS TTads who attended their initial session and were also discharged between 1 April 2013 and 18 December 2023, marking their first episode of care with the service. The starting point of 2013 was selected due to improvements in the collection of sexual orientation data, minimizing the risk of sampling bias from earlier, less reliable data.

Patients were included if they completed at least two therapy sessions and were discharged from their episode of care, indicating they were no longer receiving treatment, in line with NHS Digital reporting guidance (NHS Digital, 2021). Also, participants had to meet the clinical criteria (termed as “caseness” by services) for depression or anxiety before treatment (see Table 1 in the Supplementary Materials), and sexual orientation data needed to be recorded as heterosexual, lesbian, gay, bisexual, unsure, or ‘declined to answer’ pretreatment.

Patients were excluded from the study if they did not meet the caseness thresholds for depression or anxiety disorders before treatment or if they had a primary diagnosis not addressed by TTads’ evidence-based psychological therapies, such as schizophrenia or alcohol dependency. Also excluded were individuals under 18 years of age at the time of referral and those without recorded sexual orientation or gender before treatment.

### Measures

At each clinical appointment, services collect data on depression, anxiety symptoms, and work and social functioning, with approximately 99% of pre- and posttreatment data completed (Clark, 2018). Table 1 (in the Supplementary Materials) outlines the self-report measures and service variables included in the analyses.

Self-reported sexual orientation was recorded as heterosexual, gay/lesbian, or bisexual. Gender was recorded as male or female. To improve the differentiation of sexual minority groups, analyses were stratified by gender, given that gay typically refers to men and lesbians to women. Participants who reported being “unsure” of their sexual orientation or who “declined to answer” were excluded from the main analyses but retained in the dataset and included in sensitivity analyses.

### Data analysis plan

#### Primary outcome

‘Reliable recovery’ is defined as meeting the service-specified criteria for both ‘recovery’ and ‘reliable improvement’. Recovery is defined as starting treatment above the threshold for ‘caseness’ (i.e. the point above which someone would likely meet diagnostic criteria for the measured disorder) on either the Patient Health Questionnaire-9 (PHQ-9; Kroenke, Spitzer, & Williams, 2001) or Generalized Anxiety Disorder 7-item scale (GAD-7; Spitzer, Kroenke, Williams, & Löwe, 2006) (or an anxiety disorder-specific measure [ADSM]) or both, and then scoring below the threshold on *both measures* at the last attended treatment session.

Additionally, patients must experience reliable improvement, that is, the reduction in symptom measure scores from the first to last attended appointment is larger than the threshold for the measurement error on the given symptom scale (see secondary outcomes section). The threshold for caseness on the PHQ-9 is a score of 10 or higher. The GAD-7 threshold is eight or higher (further details, including thresholds for reliable improvement and caseness thresholds for ADSMs, are presented in Tables 1 and 2 in the Supplementary Materials).

#### Secondary outcomes

- Depressive symptoms (PHQ-9 scores) and anxiety symptoms (GAD-7) scores. These are pre-post change scores using mean differences.
- ‘Reliable Improvement’: a reduction in symptoms from the first to the last attended treatment session beyond the threshold for the measurement error on either the PHQ-9 or GAD-7 (or ADSM), or both, and no reliable deterioration reported on either symptom measure. A reduction of ≥6 points on the PHQ-9 or ≥ 4 points on the GAD-7 indicates improvement. For the criteria of reliable change on the ADSMs, refer to Table 1 in the Supplementary Materials.
- ‘Engagement’: the proportion of attended treatment sessions versus the total number of sessions offered, excluding sessions cancelled by the service or clinician but including those cancelled or not attended by the patient.
- ‘Attrition’: whether a patient discontinued treatment after completing two or more sessions, as reported by the treating clinician. Patients reported to have declined treatment or those referred elsewhere are removed from the analysis.

#### Confounders

Sociodemographic confounders included age at referral, ethnicity, and the index of multiple deprivation (IMD) decile, which ranks Lower layer Super Output Areas (LSOAs) in England by relative deprivation (Office for National Statistics, 2021).

These factors vary within sexual orientation groups, including differences in identification and expression, and are associated with therapy outcomes. Age may be relevant due to generational shifts, with younger individuals more likely to disclose their sexual identity earlier (Meyer et al., 2021). Age has also been linked to therapy outcomes (Cuijpers et al., 2020). Ethnicity may also influence sexual orientation disclosure patterns (Aranda et al., 2015), and ethnic differences have been associated with therapy outcomes (Amati et al., 2023). Socioeconomic disadvantage varies within sexual orientation groups; for example, LGBTQ individuals are considered to be at a higher risk of homelessness (UK Government, 2024). Socioeconomic deprivation has also been linked to poorer therapy outcomes (Finegan, Firth, & Delgadillo, 2020).

Clinical confounders included pretreatment depression and anxiety severity (PHQ-9, GAD-7), psychotropic medication use, long-term health conditions, personal functioning (Work and Social Adjustment Scale, items 2–5; Mundt, Marks, Shear, & Greist, 2002), ICD-10 diagnosis, and employment status.

These factors vary within sexual orientation groups, shaping lived experiences, and are associated with therapy outcomes. Depression, anxiety, and functional impairment levels differ among LGB individuals (Rimes et al., 2019), as do rates of psychotropic medication use (Bränström, Hatzenbuehler, Tinghög, & Pachankis, 2018) and chronic health conditions (Saunders et al., 2021). Greater baseline severity and comorbidity predict poorer treatment response (Buckman et al., 2021), while psychotropic medication use is associated with treatment effectiveness (Cuijpers et al., 2014). In addition, impairments in social functioning, unemployment, and long-term health conditions have been associated with worse psychological outcomes (Barnett et al., 2023; Buckman et al., 2023; Delgadillo, Dawson, Gilbody, & Böhnke, 2017).

### Data handling and data management

#### Missing data

Multiple imputations with chained equations (MICE) (Royston & White, 2011) were used to impute values for all variables listed in Table 1 (in the Supplementary Materials), creating 50 datasets with imputed values. Sexual orientation data were excluded from imputation because it was the key predictor for the analysis, and imputing it could lead to biased results if the missingness was not random. The effects of the imputation were evaluated through sensitivity analyses using only complete case data.

#### Plan of analysis

We estimated the differences in therapy outcomes by sexual orientation. For each gender, we used heterosexual groups as the reference group and compared outcomes with each sexual minority group. Thus, comparisons included lesbian women versus heterosexual women, bisexual women versus heterosexual women, gay men versus heterosexual men, and bisexual men versus heterosexual men.

Multilevel logistic regressions were employed for the primary outcome, while appropriate regression models (multilevel linear or logistic) were used for secondary outcomes. A random intercept for the service was incorporated to account for variations between services and address the clustered nature of the data.

The minimally adjusted model compared heterosexual and sexual minority groups, controlling only for pretreatment outcome measures (PHQ-9 and GAD-7). The fully adjusted model included additional controls for sociodemographic and clinical factors. The supplement material provides two further adjusted models for treatment, service and cohort factors (Tables 5 and 6).

To explore how robust the findings were regarding sexual orientation reporting, we conducted a sensitivity analysis to assess whether therapy outcomes differed between patients who indicated their sexual orientation (heterosexual, gay, lesbian, and bisexual) and those who did not (those who were unsure or declined to answer).

All analyses were conducted using Stata version 18 (StataCorp, 2023).

#### Service user and stakeholder involvement

LGB service users were consulted during the project’s development, and their support was given for its implementation. They emphasized that, in the case of negative findings, the service should focus on making improvements rather than disclosing these findings to service users. Also, meetings were held with LGB service users and NHS TTad clinicians to review and interpret the results collaboratively. In total, 12 individuals participated in the consultations.

## Results

**Figure 1.**
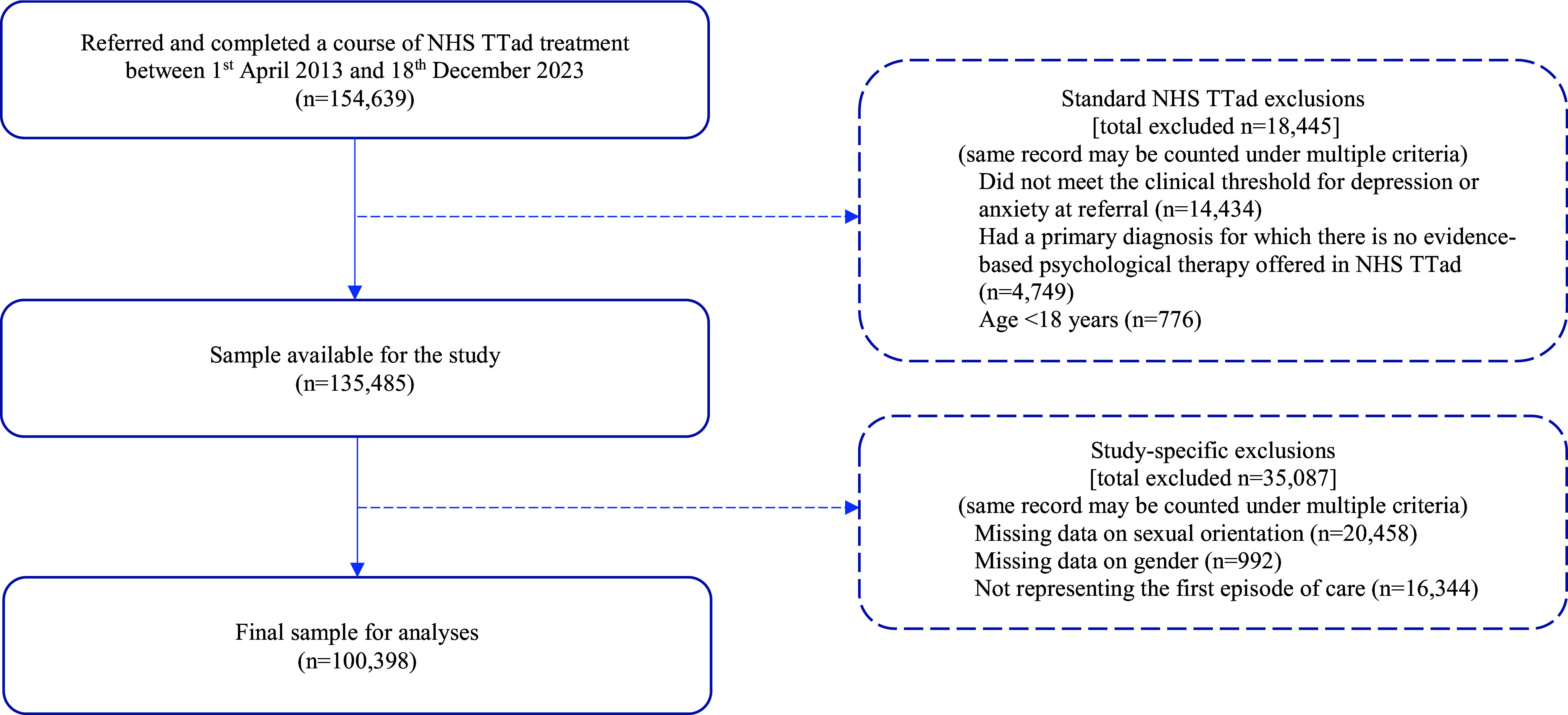
Flowchart – inclusion and exclusion of data and reasons.

A total of 154,639 individuals were referred and completed a course of NHS Talking Therapies treatment between April 2013 and December 2023. Of these, 135,485 met standard NHS Talking Therapies inclusion criteria. Furthermore, 35,087 were excluded due to missing sexual orientation data, gender not recorded, or not representing their first episode of care. This resulted in a final analytic sample of 100,389 patients (see Figure 1 for a flowchart of inclusion and exclusion of data and reasons).

Of the 94,239 individuals who indicated their sexual orientation, 557 were bisexual men (0.6%), 2,725 were gay men (2.9%), 26,017 were heterosexual men (27.6%), 2,867 were bisexual women (2.4%), 1,273 were lesbian women (1.4%), and 60,799 were heterosexual women (64.5%). Additionally, 1,185 patients (1.2% of the total sample) were unsure about their sexual orientation, and 4,975 patients (5.0% of the total sample) declined to answer and were included in sensitivity analyses.

Table 3 presents the sociodemographic, clinical characteristics, and treatment-related variables by sexual orientation for men and Table 4 for women. For those “unsure” or declined to answer, the Supplementary Materials includes Tables 7 and 8 for women and men, respectively. Proportions of missing data are also reported in each table.

**Table 3.** Sociodemographic, clinical characteristics, and treatment variables for bisexual, gay, and heterosexual men

Variable	Bisexual men, N (%) or Mean (SD)	Gay men, N (%) or Mean (SD)	Heterosexual men, N (%) or Mean (SD)
Total (N)	557	2,725	26,017
Socio-demographics			
Age	30.4 (10.7)	35.6 (11.7)	38.4 (13.6)
Ethnicity			
White	384 (68.9)	2,158 (79.2)	16,790 (64.5)
Mixed	43 (7.7)	166 (6.1)	1,408 (5.4)
Asian	62 (11.1)	139 (5.1)	3,726 (14.3)
Black	40 (7.2)	96 (3.5)	2,453 (9.4)
Chinese	6 (1.1)	35 (1.3)	167 (0.6)
Other	18 (3.2)	94 (3.4)	1,069 (4.1)
Missing	4 (0.7)	37 (1.4)	404 (1.6)
Employment status			
Employed	334 (60.0)	1,778 (65.2)	15,465 (59.4)
Unemployed	56 (10.1)	257 (9.4)	2,439 (9.4)
Student	77 (13.8)	212 (7.8)	1,468 (5.6)
Long-term sick	22 (3.9)	100 (3.7)	2,023 (7.8)
Homemaker	3 (0.5)	7 (0.3)	173 (0.7)
Not seeking work	25 (4.5)	92 (3.4)	1,973 (7.6)
Volunteer	2 (0.4)	11 (0.4)	102 (0.4)
Retired	7 (1.3)	61 (2.2)	1,138 (4.4)
Missing	31 (5.6)	207 (7.6)	1,236 (4.8)
IMD Decile			
1 (Most deprived)	27 (4.8)	176 (6.5)	1,844 (7.1)
2	107 (19.2)	503 (18.5)	5,314 (20.4)
3	99 (17.8)	420 (15.4)	4,210 (16.2)
4	55 (9.9)	307 (11.3)	3,013 (11.6)
5	38 (6.8)	199 (7.3)	2,240 (8.6)
6	50 (9.0)	189 (6.9)	2,117 (8.1)
7	17 (3.1)	102 (3.7)	1,379 (5.3)
8	19 (3.4)	96 (3.5)	1,366 (5.3)
9	6 (1.1)	34 (1.2)	520 (2.0)
10 (Least deprived)	0 (0.0)	3 (0.1)	198 (0.8)
Missing	139 (25.0)	696 (25.5)	3,816 (14.7)
Clinical characteristics			
First session PHQ–9 score	15.1 (5.5)	14.5 (5.6)	15.2 (5.7)
First session GAD–7	13.2 (4.4)	13.7 (4.5)	13.7 (4.5)
Psychotropic medication			
Prescribed not taking	22 (3.9)	193 (7.1)	1,429 (5.5)
Prescribed and taking	170 (30.5)	938 (34.4)	8,812 (33.9)
Not prescribed	329 (59.1)	1,457 (53.5)	14,322 (55.0)
Missing	36 (6.5)	137 (5.0)	1,454 (5.6)
Long-term health condition status			
No	264 (47.4)	1,339 (49.1)	14,018 (53.9)
Yes	136 (24.4)	651 (23.9)	6,899 (26.5)
Missing	157 (28.2)	735 (27.0)	5,100 (19.6)
Personal functioning (sum of items 2–5 on the WSAS)	15.8 (6.9)	15.0 (7.1)	15.7 (7.7)
Problem descriptor			
Depression	258 (46.3)	1,089 (40.0)	12,390 (47.6)
Mixed anxiety and depression	22 (3.9)	106 (3.9)	1,226 (4.7)
GAD	82 (14.7)	495 (18.2)	3,403 (13.1)
OCD	12 (2.2)	71 (2.6)	568 (2.2)
PTSD	17 (3.1)	57 (2.1)	959 (3.7)
Social phobia	29 (5.2)	181 (6.6)	1,140 (4.4)
Other phobia or panic disorder	25 (4.5)	133 (4.9)	1,452 (5.6)
Anxiety disorder not otherwise specified	2 (0.4)	10 (0.4)	542 (2.1)
Missing	110 (19.7)	583 (21.4)	4,337 (16.7)
Weeks between referral and assessment	3.1 (2.9)	3.1 (3.2)	3.0 (3.8)
Weeks between assessment and entering treatment	9.8 (9.5)	9.3 (8.9)	9.0 (8.7)
Treatment-related variables			
Number of attended sessions	8.0 (4.7)	8.0 (4.8)	7.6 (4.8)
Main treatment intensity			
Low intensity	242 (43.4)	1,182 (43.4)	12,366 (47.5)
High intensity	295 (53.0)	1,442 (52.9)	12,536 (48.2)
Missing	20 (3.6)	101 (3.7)	1,115 (4.3)
End of treatment factors			
Reliable recovery			
No	280 (50.3)	1,248 (45.8)	13,609 (52.3)
Yes	276 (49.6)	1,472 (54.0)	12,351 (47.5)
Missing	1 (0.2)	5 (0.2)	57 (0.2)
Reliable improvement			
No	160 (28.7)	691 (25.4)	7,907 (30.4)
Yes	397 (71.3)	2,034 (74.6)	18,110 (69.6)
Attrition			
No	397 (71.3)	1,916 (70.3)	16,647 (64.0)
Yes	111 (19.9)	604 (22.2)	7,044 (27.1)
Missing	49 (8.8)	205 (7.5)	2,326 (8.9)
Proportion of offered sessions attended	0.84 (0.2)	0.84 (0.2)	0.821 (0.2)
PHQ–9 change score	5.8 (5.9)	6.4 (6.2)	5.9 (6.4)
GAD–7 change score	5.3 (5.2)	6.3 (5.7)	5.4 (5.7)
PHQ–9 end score	9.2 (6.2)	8.2 (6.0)	9.3 (6.8)
GAD–7 end score	7.8 (5.2)	7.4 (5.3)	8.2 (5.8)

**Table 4.** Sociodemographic, clinical characteristics, and treatment variables for bisexual, lesbian, and heterosexual women

Variable	Bisexual women, N (%) or mean (SD)	Lesbian women, N (%) or mean (SD)	Heterosexual women, N (%) or mean (SD)
Total (N)	2,867	1,273	60,799
Socio-demographics			
Age	27.1 (7.1)	33.1 (11.6)	37.1 (13.6)
Ethnicity			
White	2,069 (72.2)	964 (75.7)	37,858 (62.3)
Mixed	290 (10.1)	103 (8.1)	4,035 (6.6)
Asian	167 (5.8)	47 (3.7)	7,554 (12.4)
Black	189 (6.6)	104 (8.2)	7,323 (12.0)
Chinese	46 (1.6)	12 (0.9)	619 (1.0)
Other	83 (2.9)	30 (2.4)	2,559 (4.2)
Missing	23 (0.8)	13 (1.0)	856 (1.4)
Employment status			
Employed	1,734 (60.5)	829 (65.1)	34,411 (56.6)
Unemployed	172 (6.0)	95 (7.5)	4,618 (7.6)
Student	560 (19.5)	132 (10.4)	4,616 (7.6)
Long-term sick	54 (1.9)	53 (4.2)	4,499 (7.4)
Homemaker	27 (0.9)	14 (1.1)	3,267 (5.4)
Not seeking work	60 (2.1)	57 (4.5)	3,434 (5.6)
Volunteer	4 (0.1)	1 (0.1)	306 (0.5)
Retired	6 (0.2)	16 (1.3)	2,625 (4.3)
Missing	250 (8.7)	76 (6.0)	3,023 (5.0)
IMD decile			
1 (Most deprived)	180 (6.3)	90 (7.1)	4,452 (7.3)
2	548 (19.1)	253 (19.9)	12,349 (20.3)
3	439 (15.3)	207 (16.3)	9,890 (16.3)
4	284 (9.9)	136 (10.7)	7,232 (11.9)
5	201 (7.0)	84 (6.6)	5,173 (8.5)
6	158 (5.5)	77 (6.0)	4,617 (7.6)
7	114 (4.0)	54 (4.2)	3,068 (5.0)
8	80 (2.8)	49 (3.8)	3,014 (5.0)
9	35 (1.2)	19 (1.5)	1,156 (1.9)
10 (Least deprived)	8 (0.3)	1 (0.1)	399 (0.7)
Missing	820 (28.6)	303 (23.8)	9,449 (15.6)
Clinical characteristics			
First session PHQ–9 score	14.6 (5.2)	14.9 (5.5)	15.1 (5.6)
First session GAD–7	13.1 (4.4)	13.8 (4.4)	14.1 (4.4)
Psychotropic medication			
Prescribed Not taking	135 (4.7)	68 (5.3)	3,566 (5.9)
Prescribed and taking	818 (28.5)	403 (31.7)	18,816 (30.9)
Not prescribed	1,738 (60.6)	724 (56.9)	34,412 (56.6)
Missing	176 (6.1)	78 (6.1)	4,005 (6.6)
Long-term health condition status			
No	1,317 (45.9)	625 (49.1)	31,616 (52.0)
Yes	613 (21.4)	315 (24.7)	16,048 (26.4)
Missing	937 (32.7)	333 (26.2)	13,135 (21.6)
Personal functioning (sum of items 2–5 on the WSAS)	15.3 (6.5)	15.4 (7.1)	15.5 (7.7)
Problem descriptor			
Depression	1,298 (45.3)	522 (41.0)	27,185 (44.7)
Mixed anxiety and depression	80 (2.8)	53 (4.2)	3,151 (5.2)
GAD	522 (18.2)	234 (18.4)	10,531 (17.3)
OCD	77 (2.7)	46 (3.6)	1,190 (2.0)
PTSD	122 (4.3)	51 (4.0)	2,000 (3.3)
Social phobia	128 (4.5)	55 (4.3)	1,550 (2.5)
Other phobia or panic disorder	129 (4.5)	61 (4.8)	3,353 (5.5)
Anxiety disorder not otherwise specified	10 (0.3)	8 (0.6)	1,062 (1.7)
Missing	501 (17.5)	243 (19.1)	10,777 (17.7)
Weeks between referral and assessment	3.3 (4.0)	3.2 (3.4)	3.2 (3.9)
Weeks between assessment and entering treatment	9.6 (9.6)	9.5 (9.5)	9.2 (8.9)
Treatment-related variables			
Number of attended sessions	8.5 (5.1)	8.3 (5.1)	7.8 (4.9)
Main treatment intensity			
Low intensity	1,183 (41.3)	540 (42.4)	27,626 (45.4)
High intensity	1,601 (55.8)	690 (54.2)	30,679 (50.5)
Missing	83 (2.9)	43 (3.4)	2,494 (4.1)
End of treatment factors			
Reliable recovery			
No	1,519 (53.0)	642 (50.4)	32,268 (53.1)
Yes	1,346 (46.9)	628 (49.3)	28,438 (46.8)
Missing	2 (0.1)	3 (0.2)	93 (0.2)
Reliable improvement			
No	826 (28.8)	368 (28.9)	18,095 (29.8)
Yes	2,041 (71.2)	905 (71.1)	42,704 (70.2)
Attrition			
No	2,000 (69.8)	855 (67.2)	39,222 (64.5)
Yes	673 (23.5)	307 (24.1)	16,374 (26.9)
Missing	194 (6.8)	111 (8.7)	5,200 (8.6)
Proportion of offered sessions attended	0.83 (0.2)	0.83 (0.2)	0.81 (0.2)
PHQ–9 change	5.6 (5.8)	6.0 (6.4)	5.8 (6.3)
GAD–7 change	5.2 (5.5)	5.8 (5.9)	5.6 (5.9)
PHQ–9 end	9.0 (5.8)	8.9 (6.3)	9.3 (6.6)
GAD–7 end	7.9 (5.1)	8.1 (5.5)	8.5 (5.8)

### Primary outcome

The estimates reported in the text are fully adjusted for pre-treatment scores, socio-demographic, and clinical factors. The Supplementary Materials include all results from minimally adjusted, fully adjusted, and models adjusted for treatment and cohort factors (Tables 5 and 6).

### Reliable recovery

Gay men had 23% higher odds of meeting reliable recovery criteria (odds ratio [OR]: 1.23, 95% confidence interval [CI]: 1.13 to 1.34). There was little evidence of a difference between the other groups: bisexual and heterosexual men (OR: 1.12, 95% CI: 0.94 to 1.33), lesbian and heterosexual women (OR: 1.09, 95% CI: 0.97 to 1.22), or bisexual and heterosexual women (OR: 0.99, 95% CI: 0.92 to 1.07).

**Figure 2.**
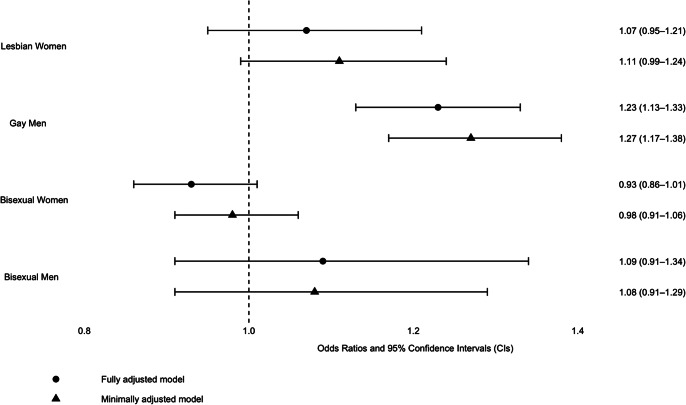
The primary outcome of reliable recovery: odds ratios for LGB vs Heterosexual (Gender-Matched). This figure presents odds ratios (AORs) and 95% confidence intervals (CIs) for reliable recovery comparing LGB groups to heterosexual individuals (reference group, dashed line). Analyses are adjusted for pretreatment scores, age, ethnicity, deprivation (IMD), medication use, health conditions, functioning, problem descriptors, and employment status.

**Figure 3.**
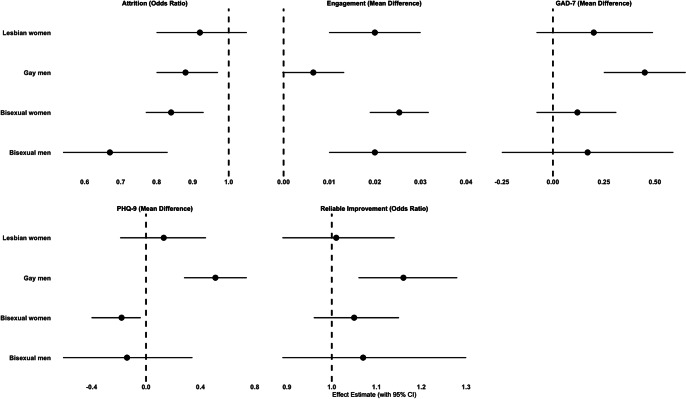
Fully adjusted secondary outcomes for LGB groups compared to the heterosexual reference group (gender-matched). This figure presents adjusted odds ratios or mean differences with 95% confidence intervals (CIs) for secondary outcomes comparing LGB groups to heterosexual patients (reference group, dashed line). Analyses are adjusted for pretreatment scores, age, ethnicity, deprivation (IMD), medication use, health conditions, functioning, problem descriptors, and employment status.

Visual summaries are provided. Figure 2 presents the primary outcome of reliable recovery, displaying minimally and fully adjusted ORs for LGB individuals compared to gender-matched heterosexual counterparts. Figure 3 illustrates fully adjusted secondary outcomes for LGB groups relative to the gender-matched heterosexual reference group.

### Secondary outcomes

#### Reliable improvement

Gay men had 16% higher odds of reliable improvement (OR: 1.16, 95% CI: 1.06 to 1.28). There was little evidence of a difference between the other groups: bisexual and heterosexual men (OR: 1.07, 95% CI: 0.89 to 1.30), lesbian and heterosexual women (OR: 1.01, 95% CI: 0.89 to 1.14), or bisexual and heterosexual women (OR: 1.05, 95% CI: 0.96 to 1.15).

#### Attrition

In absolute terms, the attrition rate was 22.2% (604/2,725) for gay men, 19.9% (111/557) for bisexual men, and 27.1% (7,044/26,017) for heterosexual men. Among women, the attrition rate was 24.1% (307/1,273) for lesbian women, 23.5% (673/2,867) for bisexual women, and 26.9% (16,374/60,799) for heterosexual women.

Gay men had 12% lower odds of attrition compared to heterosexual men (OR: 0.88, 95% CI: 0.80 to 0.97), while bisexual men had 33% lower odds (OR: 0.67, 95% CI: 0.54 to 0.83). Bisexual women had 16% lower odds of attrition compared to heterosexual women (OR: 0.84, 95% CI: 0.77 to 0.93). No evidence of a difference was observed between lesbian and heterosexual women (OR: 0.92, 95% CI: 0.80 to 1.05).

#### PHQ-9 (depression severity)

Gay men experienced greater reductions in depressive symptoms (mean difference [MD]: 0.51, 95% CI: 0.28 to 0.74). There was little evidence of differences between bisexual and heterosexual men (MD: −0.14, 95% CI: −0.16 to 0.34), lesbian and heterosexual women (MD: 0.13, 95% CI: −0.19 to 0.44), or bisexual and heterosexual women (MD: -0.18, 95% CI: −0.40 to 0.04).

#### GAD-7 (anxiety severity)

Gay men experienced greater reductions in anxiety symptoms (MD: 0.45, 95% CI: 0.25 to 0.65). Little evidence was found for the other groups: bisexual and heterosexual men (MD: 0.17, 95% CI: −0.25 to 0.59), bisexual and heterosexual women (MD: 0.12, 95% CI: −0.08 to 0.31), or lesbian and heterosexual women (MD: 0.20, 95% CI: −0.08 to 0.49).

#### Treatment engagement

Bisexual men attended more sessions than heterosexual men (an average of 8.0 sessions versus 7.6 sessions, MD: 0.02, 95% CI: 0.01 to 0.04). For gay men, who also attended an average of 8.0 sessions, there was little evidence of a difference compared to heterosexual men (MD: 0.01, 95% CI: 0.00 to 0.02).

Lesbian and bisexual women attended more sessions than heterosexual women. On average, lesbian women attended 8.3 sessions, and bisexual women attended 8.5 sessions, compared to 7.8 sessions for heterosexual women. The mean differences were 0.02 (95% CI: 0.01 to 0.03) for lesbian women and 0.03 (95% CI: 0.02 to 0.03) for bisexual women.

#### Sensitivity analyses

There was little evidence of differences in therapy outcomes for men, regardless of whether they disclosed their sexual orientation. Among women, there was evidence of differences in reliable recovery, reliable improvement, PHQ-9 change, GAD-7 change, and engagement for those who were unsure or declined to answer regarding sexual orientation compared to those who disclosed (see the Supplementary Materials, Tables 9 and 10).

Complete case models alongside the main analysis showed consistent estimates with overlapping CIs, indicating minimal sensitivity to missing data. Multiple imputation produced slightly smaller estimates without altering the overall conclusions (see the Supplementary Materials, Tables 11 and 12).

## Discussion

### Summary of key findings

This study analyzed therapy outcomes for 100,389 patients across seven London NHS TTads, focusing on differences by sexual orientation and gender. The rates of reliable recovery and reliable improvement were higher among gay men compared to heterosexual men, and the rate of attrition was lower. There were also greater reductions in depressive and anxiety symptoms for gay men compared to heterosexual men. Bisexual men and women had lower rates of attrition than heterosexual men and women, respectively. Lesbian and bisexual women, as well as bisexual men, demonstrated slightly better treatment engagement than their heterosexual counterparts. There was little evidence of differences in other therapy outcomes across these groups.

Although gay men demonstrated better outcomes than heterosexual men across several therapy metrics, the magnitude of symptom improvement was small at the individual level and should be interpreted with caution in terms of representing a meaningful differential treatment benefit. Average symptom reductions were 6.4 points on the PHQ-9 and 6.3 on the GAD-7 for gay men, compared to 5.9 and 5.4 points, respectively, for heterosexual men. The resulting mean differences – 0.51 (PHQ-9) and 0.45 (GAD-7) – were statistically significant, likely reflecting the large sample size, but fell well below established thresholds for minimum clinically important differences (MCIDs; approximately 1.9 for the PHQ-9 and 1.6 for the GAD-7; Button et al., 2015; Kounali et al., 2022).

Nonetheless, symptom change was highly variable (SD ≈ 6 points for both measures), meaning that even small shifts in group means can influence the proportion of individuals meeting clinical thresholds. In this sample, 54.0% of gay men met the reliable recovery metric, compared to 47.5% of heterosexual men – a 6.5 percentage point difference. While average improvements were small and below clinically meaningful thresholds, the higher recovery rate among gay men represents a positive group-level outcome. This illustrates how modest differences in mean symptom change can translate into meaningful variation in categorical outcomes within large-scale psychological therapy services.

### Comparison with previous literature

Our findings differ from those reported by earlier studies (Rimes et al., 2018, 2019), which identified poorer outcomes for bisexual individuals and lesbian women, with no differences observed for gay men.

A key distinction in the present study is the larger LGB sample size (*n* = 7,422) compared to the smaller samples in previous studies (*n* = 1,130 in Rimes et al., 2018 and *n* = 4,472 in Rimes et al., 2019). This 66% increase over the 2019 study and a 557% increase over the 2018 study enhances statistical power and precision, enabling more detailed subgroup analyses, particularly for bisexual individuals, who were underrepresented (e.g. 297 in Rimes et al., 2018 versus 3,424 in our study).

Regional and demographic differences may contribute to the observed discrepancies. This study was conducted in North and East London, which differs from the national cohort (Rimes et al., 2019) and may also differ slightly from the South London sample (Rimes et al., 2018). North and East London have the highest proportion of LGB residents in England (Office for National Statistics, 2021), which could influence the minority stress levels experienced by LGB individuals. Increased opportunities for contact with other LGB individuals in this region might provide social protective effects, such as fostering a sense of community and acceptance (Meyer, 2003; Rogowska & Cisek, 2024).

Our findings are broadly consistent with international studies reporting similar therapy outcomes between LGB and heterosexual patients. However, differences between our study and these studies – such as clinical context, presentation, and sampling – make direct comparison challenging. One possible explanation for this convergence is that common therapeutic factors may support similar outcomes across groups when care is delivered in an inclusive and nondiscriminatory manner.

### Consultations with LGB service users and therapists

To support the interpretation and contextualization of the results, semistructured consultations were conducted with LGB service users and therapists, comprising two group discussions and one individual meeting. This approach aligns with national guidance promoting the involvement of the public, including those with relevant lived experience, in interpreting research findings (National Institute for Health Research [NIHR], 2021). Discussions explored potential explanations for the observed quantitative patterns in engagement and outcomes. A key theme identified was a culture of openness and normalization toward therapy, alongside a strong motivation to engage with treatment within the LGB community. Service users noted that gay men may feel more at ease expressing emotions and seeking support, which could enhance engagement with therapy. Lesbian and bisexual women emphasized the importance of therapy as a safe and non-judgmental space to address issues they might not feel comfortable discussing elsewhere. Across all groups, participants highlighted the vital role of feeling accepted, understood, and supported by therapists. These factors may help explain the observed levels of engagement and comparable outcomes *despite* the high burden of mental health difficulties within these populations.

### Strengths and limitations

This study has several strengths. A large sample size improved the precision of estimates. Routinely collected NHS data reflected real-world clinical practice and outcomes. Sociodemographic and clinical variables allowed for robust covariate adjustment and accounted for confounders. Statistical analyses addressed data clustering at the service level through multilevel regression. Missing data were addressed using multiple imputation, and sensitivity was investigated through a complete case analysis. Sensitivity analyses explored the role of sexual orientation disclosure in therapy outcomes. Selection bias was minimized as it included most service users who entered the service. Using PHQ-9 and GAD-7 difference scores retains the advantages of continuous data and can improve detection of group differences, especially when adjusting for pretreatment measures.

The observational design of this study limits causal inferences about the relationship between sexual orientation and therapy outcomes. However, adjustments for baseline pretreatment scores and other potential confounders strengthen our findings.

Sexual orientation itself does not cause mental health disparities but reflects differential exposure to minority stress rooted in structural inequities. As Meyer (2003) and Hatzenbuehler (2009) argued, disparities stem not from orientation itself but from systemic marginalization – stigma, discrimination, and exclusion – that heightens risk for sexual minorities. However, the individual remains the unit of analysis: lived experiences of stress (e.g. internalized stigma, concealment) occur within an individual, even as broader systems shape these processes. Mental health outcomes are partly determined by how individuals process and navigate these stressors and available resources, rather than by their group membership alone. This study did not directly measure minority stress, limiting its ability to explain individual variation or assess its influence on therapy outcomes.

Measurement limitations should be noted. The study’s measures of sexual orientation may be affected by measurement error, as some LGB service users could have concealed their sexual orientation by identifying as heterosexual. This issue is a recognized challenge in research involving “hidden” populations (Pachankis & Bränström, 2019). Such nondifferential measurement error would likely bias results toward the null and is therefore unlikely to explain the observed improvements in response rates and reduced attrition among gay men. A binary gender classification was used in this study, as available data predominantly recorded gender as “male” or “female.” As a result, transgender, nonbinary, and other gender-diverse individuals were not separately represented in the analyses. This limitation fails to capture the complexity of gender identity and expression. Butler’s (1990) critique of normative gender categories challenges the assumption of a fixed binary, instead proposing a fluid, socially constructed understanding of gender. While this study focused on sexual orientation, gender-diverse individuals also experience elevated minority stress and mental health disparities, often shaped by the intersection of gender and sexuality (Tan, Treharne, & Ellis, 2020). Since data collection, TTads have expanded their gender categories (e.g. nonbinary, intersex), supporting more inclusive and detailed analyses of therapy outcomes in future research.

Although this study focused on treatment outcomes – defined by NHS TTad as attending two or more sessions – we conducted an exploratory, post-hoc descriptive review of early disengagement (drop-out between referral and the second session) to contextualize the findings (Supplementary Table 13). Early disengagement differed by only 1–3 percentage points between heterosexual and gay or lesbian patients, suggesting limited potential for selection bias in recovery and engagement estimates. Bisexual patients showed slightly higher disengagement (5–7 percentage points). While we cannot know how these early leavers would have responded to treatment, even assuming none recovered, the resulting shifts in recovery proportions are unlikely to be large enough to alter the interpretation of our main findings. These patterns highlight the value of further investigation into barriers to early engagement, especially for bisexual individuals.

Although we preregistered our analysis plan, we did not specify a priori hypotheses regarding differences between sexual orientation groups, as we did not have clear expectations about the direction of effects. This reflected a combination of past evidence of poorer outcomes and more recent improvements in social and clinical conditions. As such, the conclusions drawn from this study should be interpreted as descriptive rather than confirmatory.

We also acknowledge the possibility that data were not missing at random – for example, due to systematic differences in data recording practices at the service or clinician level. It is also important to note that sexual orientation data were not imputed. Although multiple imputation assumes that data are missing at random, sensitivity analyses showed consistent results between imputed and complete-case analyses, suggesting a minimal risk of bias from violations of this assumption.

### Implications for research and practice

This study focused on urban services in a region with a large LGB population. Further research in rural areas or regions with smaller LGB populations could help identify structural and social factors influencing therapy outcomes. An updated analysis of the national TTADS dataset would be valuable for assessing the generalizability of these results.

Although we found little evidence that LGB individuals experience worse therapy outcomes than their heterosexual peers, previous research suggests that LGB clients often report poorer therapy *experiences* due to therapists’ failure to address LGB-specific issues (Foy, Morris, Fernandes, & Rimes, 2019; Morris, Fernandes, & Rimes, 2022). The consultations with LGB service users that we conducted to support the interpretation of our findings reinforced the importance of addressing these concerns, even when overall treatment outcomes appear comparable. This highlights the need for a more nuanced understanding of individual variation within LGB groups, particularly for those experiencing higher minority stress, and the role of therapists in responding to these challenges.

There is, however, limited research on how minority stress changes during *routine* therapy and how these changes affect outcomes. For example, does stigma-related stress decrease as the therapeutic relationship develops, or can specific therapeutic processes, such as identity validation or emotional safety, shape its trajectory? Although the effects observed in our study were small, the finding that gay men achieved marginally better outcomes despite potential stigma-related stressors suggests that elements of the therapeutic environment may help to buffer minority stress. Identifying the factors that contribute to this effect could inform more inclusive and effective therapeutic practices for sexual minority clients.

Overall, these findings are reassuring, suggesting that LGB individuals engaging with London TTADS services achieve similar therapy outcomes to their heterosexual peers. This contrasts with earlier research indicating poorer outcomes for lesbian and bisexual women and bisexual men. While encouraging, further research is needed to establish the generalizability of these findings and explore the factors underlying these outcomes.

## Supporting information

Kent et al. supplementary material Kent et al. supplementary material

## Data Availability

There are restrictions on the availability of data used in this study per the NCEL agreement (project reference: 00519-IAPT). Data were used under license for the current study and so are not publicly available. The code used to generate these results has been archived at https://github.com/jae-suh74/ttad-outcomes-lgb/.

## Supplementary material

The supplementary material for this article can be found at http://doi.org/10.1017/S0033291725101220.

## References

[r1] Amati, F., Green, J., Kitchin, L., Watt, H., Jones, S., AlRubaye, N., … Greenfield, G. (2023). Ethnicity as a predictor of outcomes of psychological therapies for anxiety and depression: A retrospective cohort analysis. Behavioural and Cognitive Psychotherapy, 51(2), 164–173. 10.1017/S1352465822000558. 36740941

[r2] Amos, R., Manalastas, E. J., White, R., Bos, H., & Patalay, P. (2020). Mental health, social adversity, and health-related outcomes in sexual minority adolescents: A contemporary national cohort study. The Lancet Child & Adolescent Health, 4(1), 36–45. 10.1016/S2352-4642(19)30339-6. 31753807

[r3] Aranda, F., Matthews, A. K., Hughes, T. L., Muramatsu, N., Wilsnack, S. C., Johnson, T. P., & Riley, B. B. (2015). Coming out in color: Racial/ethnic differences in the relationship between level of sexual identity disclosure and depression among lesbians. Cultural Diversity and Ethnic Minority Psychology, 21(2), 247–257. 10.1037/a0037644. 25181323 PMC4345130

[r4] Ayhan, C. H. B., Bilgin, H., Uluman, O. T., Sukut, O., Yilmaz, S., & Buzlu, S. (2020). A systematic review of the discrimination against sexual and gender minority in health care settings. International Journal of Health Services, 50(1), 44–61. 10.1177/0020731419885093. 31684808

[r5] Barker, M.J. (2019). Good practice across the counselling professions 001: Gender, sexual, and relationship diversity (GSRD). British Association for Counselling and Psychotherapy. https://www.bacp.co.uk/media/5877/bacp-gender-sexual-relationship-diversity-gpacp001-april19.pdf

[r6] Barnett, P., Saunders, R., Buckman, J. E., Naqvi, S. A., Singh, S., Stott, J., … Pilling, S. (2023). The association between trajectories of change in social functioning and psychological treatment outcome in university students: A growth mixture model analysis. Psychological Medicine, 53(14), 6848–6858. 10.1017/S0033291723000363. 36876490 PMC10600814

[r7] Bar-Sella, A., Nof, A., Baucom, B. R., Goldstein, P., Romanov, S., Shpakouskaya, I., Kaplun, D., & Zilcha-Mano, S. (2023). The prognostic role of emotion regulation dynamics in the treatment of major depressive disorder. Journal of Consulting and Clinical Psychology, 91(12), 744–749. 10.1037/ccp0000835. 37616125

[r8] Beattie, S., & Laville, A. (2024). LGBTQ+ Positive practice guide. NHS Talking Therapies for Anxiety and Depression. https://lgbt.foundation/wp-content/uploads/2024/07/LGBTQ-Positive-Practice-Guide-20248_DIGITAL_FINAL.pdf.

[r9] Beard, C., Kirakosian, N., Silverman, A. L., Winer, J. P., Wadsworth, L. P., & Björgvinsson, T. (2017). Comparing treatment response between LGBQ and heterosexual individuals attending a CBT-and DBT-skills-based partial hospital. Journal of Consulting and Clinical Psychology, 85(12), 1171. 10.1037/ccp0000251. 29189033

[r10] Bezahler, A., Kuckertz, J. M., Schreck, M., Narine, K., Dattolico, D., & Falkenstein, M. J. (2022). Examination of outcomes among sexual minorities in treatment for obsessive-compulsive and related disorders. Journal of Obsessive-Compulsive and Related Disorders, 33, 100724. 10.1016/j.jocrd.2022.100724. 37220532 PMC10201929

[r11] Donahue, J. M., DeBenedetto, A. M., Wierenga, C. E., Kaye, W. H., & Brown, T. A. (2020). Examining day hospital treatment outcomes for sexual minority patients with eating disorders. International Journal of Eating Disorders, 53(10), 1657–1666. 10.1002/eat.23362. 32808329

[r12] Bränström, R., Hatzenbuehler, M. L., Tinghög, P., & Pachankis, J. E. (2018). Sexual orientation differences in outpatient psychiatric treatment and antidepressant usage: Evidence from a population-based study of siblings. European Journal of Epidemiology, 33, 591–599. 10.1007/s10654-018-0411-y. 29766438 PMC5995973

[r13] Brooks, V. R. (1981). Minority stress and lesbian women. Free Press.

[r14] Buckman, J. E., Saunders, R., Cohen, Z. D., Barnett, P., Clarke, K., Ambler, G., … Pilling, S. (2021). The contribution of depressive ‘disorder characteristics’ to determinations of prognosis for adults with depression: An individual patient data meta-analysis. Psychological Medicine, 51(7), 1068–1081. 10.1017/S0033291721001367. 33849685 PMC8188529

[r15] Buckman, J. E., Stott, J., Main, N., Antonie, D. M., Singh, S., Naqvi, S. A., … Saunders, R. (2023). Understanding the psychological therapy treatment outcomes for young adults who are not in education, employment, or training (NEET), moderators of outcomes, and what might be done to improve them. Psychological Medicine, 53(7), 2808–2819. 10.1017/S0033291721004773. 37449486 PMC10235648

[r16] Butler, J. (1990). Gender trouble: Feminism and the subversion of identity. Routledge.

[r17] Button, K. S., Kounali, D., Thomas, L., Wiles, N. J., Peters, T. J., Welton, N. J., … Lewis, G. (2015). Minimal clinically important difference on the Beck depression inventory-II according to the patient’s perspective. Psychological Medicine, 45(15), 3269–3279. 10.1017/S0033291715001270. 26165748 PMC4611356

[r18] Clark, D. M. (2011). Implementing NICE guidelines for the psychological treatment of depression and anxiety disorders: The IAPT experience. International Review of Psychiatry, 23(4), 318–327. 10.3109/09540261.2011.606803. 22026487 PMC3212920

[r19] Clark, D. M. (2018). Realizing the mass public benefit of evidence-based psychological therapies: The IAPT program. Annual Review of Clinical Psychology, 14(1), 159–183. 10.1146/annurev-clinpsy-050817-084833. PMC5942544 29350997

[r20] Cuijpers, P., Karyotaki, E., Eckshtain, D., Ng, M. Y., Corteselli, K. A., Noma, H., … Weisz, J. R. (2020). Psychotherapy for depression across different age groups: A systematic review and meta-analysis. JAMA Psychiatry, 77(7), 694–702. 10.1001/jamapsychiatry.2020.0164. 32186668 PMC7081149

[r21] Cuijpers, P., Sijbrandij, M., Koole, S. L., Andersson, G., Beekman, A. T., & Reynolds, C. F. (2014). Adding psychotherapy to antidepressant medication in depression and anxiety disorders: A meta-analysis. Focus, 12(3), 347–358. 10.1002/wps.20089. PMC3918025 24497254

[r22] Cuijpers, P., Miguel, C., Ciharova, M., Harrer, M., Basic, D., Cristea, I. A., … Karyotaki, E. (2024). Absolute and relative outcomes of psychotherapies for eight mental disorders: A systematic review and meta-analysis. World Psychiatry, 23(2), 267–275. 38727072 10.1002/wps.21203 PMC11083862

[r23] Delgadillo, J., Dawson, A., Gilbody, S., & Böhnke, J. R. (2017). Impact of long-term medical conditions on the outcomes of psychological therapy for depression and anxiety. The British Journal of Psychiatry, 210(1), 47–53. 10.1192/bjp.bp.116.189027. 27856421

[r24] Feinstein, B. A. (2020). The rejection sensitivity model as a framework for understanding sexual minority mental health. Archives of Sexual Behavior, 49(7), 2247–2258. 10.1007/s10508-019-1428-3. 31286339 PMC8714401

[r25] Finegan, M., Firth, N., & Delgadillo, J. (2020). Adverse impact of neighbourhood socioeconomic deprivation on psychological treatment outcomes: The role of area-level income and crime. Psychotherapy Research, 30(4), 546–554. 10.1080/10503307.2019.1649500. 31366303

[r26] Foy, A. A., Morris, D., Fernandes, V., & Rimes, K. A. (2019). LGBQ+ adults’ experiences of improving access to psychological therapies and primary care counselling services: Informing clinical practice and service delivery. The Cognitive Behaviour Therapist, 12, e42. 10.1017/S1754470X19000291.

[r27] Hatzenbuehler, M. L. (2009). How does sexual minority stigma “get under the skin”? A psychological mediation framework. Psychological Bulletin, 135(5), 707. 10.1037/a0016441. 19702379 PMC2789474

[r28] Huchet-Bodet, A. M. A., Albakri, M., & Smith, N. S. (2019). *Attitudes to equalities: The Retrieved from British Social Attitudes Survey 2017.* https://assets.publishing.service.gov.uk/media/5c5401e2e5274a491a41388a/Attitudes-equality-social-attitudes-survey-2017a.pdf.

[r29] Jones, N. P., Siegle, G. J., & Thase, M. E. (2008). Effects of rumination and initial severity on remission to cognitive therapy for depression. Cognitive Therapy and Research, 32, 591–604. 10.1007/s10608-008-9191-0. PMC3864875 24353355

[r30] King, M., Semlyen, J., Tai, S. S., Killaspy, H., Osborn, D., Popelyuk, D., & Nazareth, I. (2008). A systematic review of mental disorder, suicide, and deliberate self harm in lesbian, gay and bisexual people. BMC Psychiatry, 8, 1–17. 10.1186/1471-244X-8-70. 18706118 PMC2533652

[r31] Kounali, D., Button, K. S., Lewis, G., Gilbody, S., Kessler, D., Araya, R., & Lewis, G. (2022). How much change is enough? Evidence from a longitudinal study on depression in UK primary care. Psychological Medicine, 52(10), 1875–1882. 10.1017/S0033291720003700. 33138872 PMC9340848

[r32] Kroenke, K., Spitzer, R. L., & Williams, J. B. W. (2001). The PHQ-9: Validity of a brief depression severity measure. Journal of General Internal Medicine, 16, 606–613. 10.1046/j.1525-1497.2001.016009606.x. 11556941 PMC1495268

[r33] Maji, S., Yadav, N., & Gupta, P. (2023). LGBTQ+ in workplace: A systematic review and reconsideration. Equality, Diversity and Inclusion: An International Journal, 43(2), 313–360. 10.1108/EDI-02-2022-0049.

[r34] Marriage (Same-Sex Couples) Act 2013, c. 30. https://www.legislation.gov.uk/ukpga/2013/30/contents/enacted.

[r35] Meads, C., Hunt, R., Martin, A., & Varney, J. (2019). A systematic review of sexual minority women’s experiences of health care in the UK. International Journal of Environmental Research and Public Health, 16(17), 3032. 10.3390/ijerph16173032. 31438599 PMC6747244

[r36] Meyer, I. H. (2003). Prejudice, social stress, and mental health in lesbian, gay, and bisexual populations: Conceptual issues and research evidence. Psychological Bulletin, 129(5), 674. 10.1037/0033-2909.129.5.674. 12956539 PMC2072932

[r37] Meyer, I. H., Russell, S. T., Hammack, P. L., Frost, D. M., & Wilson, B. D. (2021). Minority stress, distress, and suicide attempts in three cohorts of sexual minority adults: A US probability sample. PLoS One, 16(3), e0246827. 10.1371/journal.pone.0246827. 33657122 PMC7928455

[r38] Morris, D. D., Fernandes, V., & Rimes, K. A. (2022). Sexual minority service user perspectives on mental health treatment barriers to care and service improvements. International Review of Psychiatry, 34(3–4), 230–239. 10.1080/09540261.2022.2051445. 36151833

[r39] Mundt, J. C., Marks, I. M., Shear, M. K., & Greist, J. H. (2002). The work and social adjustment scale: A simple measure of impairment in functioning. British Journal of Psychiatry, 180, 461–464. 10.1192/bjp.180.5.461. 11983645

[r40] NHS Digital. (2021). *Implementation tools and guidance: Improving access to psychological therapies (IAPT).* https://digital.nhs.uk/data-and-information/data-collections-and-data-sets/data-sets/improving-access-to-psychological-therapies-data-set/implementing-iapt-tools-and-guidance.

[r41] National Institute for Health Research. (2021). *Briefing notes for researchers: Public involvement in NHS, health and social care research.* https://www.nihr.ac.uk/briefing-notes-researchers-public-involvement-nhs-health-and-social-care-research.

[r42] NHS Digital. (2023). *NHS Talking Therapies for anxiety and depression: Annual report, 2023–24.* https://digital.nhs.uk/data-and-information/publications/statistical/nhs-talking-therapies-for-anxiety-and-depression-annual-reports/2023-24.

[r43] Office for National Statistics. (2021). *Census 2021 geographies.* Retrieved March 21, 2024, from https://www.ons.gov.uk/methodology/geography/ukgeographies/censusgeographies/census2021geographies#lower-layer-super-output-areas-lsoas

[r44] Office of National Statistics. (2022). Sexual orientation, UK: 2020. https://www.ons.gov.uk/peoplepopulationandcommunity/culturalidentity/sexuality/bulletins/sexualidentityuk/2020

[r45] Pachankis, J. E., & Bränström, R. (2019). How many sexual minorities are hidden? Projecting the size of the global closet with implications for policy and public health. PLoS One, 14(6), e0218084. 10.1371/journal.pone.0218084. 31194801 PMC6564426

[r46] Pitman, A., Marston, L., Lewis, G., Semlyen, J., McManus, S., & King, M. (2022). The mental health of lesbian, gay, and bisexual adults compared with heterosexual adults: Results of two nationally representative English household probability samples. Psychological Medicine, 52(15), 3402–3411. 10.1017/S0033291721000052. 33592165

[r47] Plöderl, M., & Tremblay, P. (2015). Mental health of sexual minorities. A systematic review. International Review of Psychiatry, 27(5), 367–385. 10.3109/09540261.2015.1083949. 26552495

[r48] Plöderl, M., Kunrath, S., Cramer, R. J., Wang, J., Hauer, L., & Fartacek, C. (2017). Sexual orientation differences in treatment expectation, alliance, and outcome among patients at risk for suicide in a public psychiatric hospital. BMC Psychiatry, 17, 1–13. 10.1186/s12888-017-1337-8. 28506219 PMC5433065

[r49] Plöderl, M., Mestel, R., & Fartacek, C. (2022). Differences by sexual orientation in treatment outcome and satisfaction with treatment among inpatients of a German psychiatric clinic. PLoS One, 17(1). 10.1371/journal.pone.0262928. PMC8782353 35061835

[r50] Richards, C., Gibson, S., Jamieson, R., & Lenihan, P. (2019). Guidelines for psychologists working with gender, sexuality and relationship diversity: For adults and young people (aged 18 and over) (2nd ed). The British Psychological Society.

[r51] Rimes, K. A., Broadbent, M., Holden, R., Rahman, Q., Hambrook, D., Hatch, S. L., & Wingrove, J. (2018). Comparison of treatment outcomes between lesbian, gay, bisexual and heterosexual individuals receiving a primary care psychological intervention. Behavioural and Cognitive Psychotherapy, 46(3), 332–349. 10.1017/S1352465817000583. 28978366

[r52] Rimes, K. A., Ion, D., Wingrove, J., & Carter, B. (2019). Sexual orientation differences in psychological treatment outcomes for depression and anxiety: National cohort study. Journal of Consulting and Clinical Psychology, 87(7), 577. 10.1037/ccp0000416. 31219292

[r53] Rogowska, A. M., & Cisek, A. (2024). Minority stress, perceived social support, and depression in people diverse in sexual and gender minority status. Psychology & Sexuality, 15(4), 679–693. 10.1080/19419899.2024.2333858.

[r54] Royston, P., & White, I. R. (2011). Multiple imputation by chained equations (MICE): Implementation in Stata. Journal of Statistical Software, 45(4), 1–20. 10.18637/jss.v045.i04.

[r55] Saunders, R., Cape, J., Leibowitz, J., Aguirre, E., Jena, R., Cirkovic, M., … Buckman, J. E. (2020). Improvement in IAPT outcomes over time: Are they driven by changes in clinical practice? The Cognitive Behaviour Therapist, 13, e16. 10.1017/S1754470X20000173. 33613689 PMC7872157

[r56] Saunders, C. L., MacCarthy, S., Meads, C., Massou, E., Mant, J., Saunders, A. M., & Elliott, M. N. (2021). Long-term conditions among sexual minority adults in England: Evidence from a cross-sectional analysis of responses to the English GP patient survey. BJGP Open, 5(5). 10.3399/BJGPO.2021.0067. PMC8596314 34465579

[r57] Spitzer, R. L., Kroenke, K., Williams, J. B. W., & Löwe, B. (2006). A brief measure for assessing generalized anxiety disorder. Archives of Internal Medicine, 166(10), 1092. 10.1001/archinte.166.10.1092. 16717171

[r58] StataCorp. (2023). *Stata statistical software: Release 18* [Software]. College Station, TX: StataCorp LLC.

[r59] Tan, K. K. H., Treharne, G. J., Ellis, S. J., Schmidt, J. M., & Veale, J. F. (2020). Gender minority stress: A critical review. Journal of Homosexuality, 67(10), 1471–1489. 10.1080/00918369.2019.1591789. 30912709

[r60] Teo, C., Metheny, N., & Chum, A. (2022). Family support modifies the effect of changes to same-sex marriage legislation on LGB mental health: Evidence from a UK cohort study. European Journal of Public Health, 32(1), 35–40. 10.1093/eurpub/ckab139. 34448847 PMC9090167

[r61] UK Government. (2024). Lesbian, gay, bisexual and transgender people’s experiences of homelessness. GOV.UK. https://www.gov.uk/government/publications/lgbt-peoples-experiences-of-homelessness/lesbian-gay-bisexual-and-transgender-peoples-experiences-of-homelessness

[r62] World Health Organization. (1993). The ICD-10 classification of mental and behavioural disorders: Diagnostic criteria for research (Vol. 2). World Health Organization.

