# Supplementary material for: Psychological therapy outcomes by sexual orientation and gender: a retrospective cohort study
Source: Psychol Med. 2025 Jul 24;55:e212. doi: 10.1017/S0033291725101220 (PMC12315664; doi:10.1017/S0033291725101220)
Supplement: Kent et al. supplementary material [file S0033291725101220sup001.docx]

### Online Supplementary Materials

### **Table 1.** Data variables with details

| Category | Assessment Tool/Method | Details and Thresholds |
| --- | --- | --- |
| Anxiety | *Generalized Anxiety Disorder 7-item (GAD-7); (Spitzer, Kroenke, Williams, & Löwe, 2006)* | A self-report questionnaire comprising seven questions that assess the severity of generalized anxiety disorder symptoms. GAD-7: Score ≥ 8 indicates clinical caseness. |
| Cohort Factors | *Service Data* | Month and Year of first therapy session |
| Demographics | *Self-Report at Referral* | Includes sexual orientation, gender, age, IMD decile, and ethnicity based on UK census codes. |
| Depression | *Patient Health Questionnaire 9-item (PHQ-9); (Kronke, Spitzer, & Williams, 2001)* | A self-report questionnaire consisting of nine questions used to assess the severity of depression symptoms. Score ≥ 10 indicates clinical caseness. |
| Employment Status | *Patient Self-Report* | Categories: employed, unemployed, Student, Long-term Sick, Homemaker, not seeking work, volunteer, retired |
| Long-term Health Conditions | *Patient Self-report* | Asks about the presence of any long-term health conditions; Specific conditions not detailed |
| Psychotropic Medication | *Patient Self-report* | Categories: prescribed and taking, prescribed and not taking, not prescribed |
| Personal Functioning | *Work and Social Adjustment Scale (Mundt et al., 2002)* | Assesses home management, social activities, private leisure, and close relationships. Items 2-5 in sum. |
| Problem Descriptor | *ICD-10 Codes* | Matches patients to treatments; Covers various mental health conditions e.g., depressive disorder |
| Treatment Factors | *Service Data* | Low-intensity/high treatment intensity, number of sessions, timeframes between referral and first treatment session. |

### **Table 2**. Anxiety disorder-specific measures (ADSMs), thresholds and reliable change.

| **Disorder** | **Specific Measure** | **Threshold for Caseness** | **Change for Reliable Change** |
| --- | --- | --- | --- |
| Agoraphobia | Mobility Inventory (Chambless, et al., 1985) | Scores of 2.3 and above | Change of 0.73 |
| Health Anxiety | Health Anxiety Inventory (Salkovskis et al., 2002) | Scores of 18 and above | Change of 4 |
| Obsessive Compulsive Disorder (OCD) | Obsessive Compulsive Inventory (Foa et al. 1998) | Scores of 40 and above | Change of 32 |
| Panic Disorder | Panic Disorder Severity Scale (PDSS; Shear at al., 1997) | Scores of 8 and above | Change of 5 |
| Post-Traumatic Stress Disorder (PTSD) | Impact of events Scale (IES-R; Creamer et al., 2003) | Scores of 33 and above | Change of 9 |
| Social Anxiety Disorder | Social Phobia Inventory (Connor et al., 2000) | Scores of 19 and above | Change of 10 |

**References:**

Chambless, D. L., Caputo, G. C., Jasin, S. E., Gracely, E. J., & Williams, C. (1985). The Mobility Inventory for Agoraphobia. *Behaviour Research and Therapy, 23*(1), 35–44. https://doi.org/10.1016/0005-7967(85)90140-8

Connor, K. M., Davidson, J. R. T., Churchill, L. E., Sherwood, A., Weisler, R. H., & Foa, E. B. (2000). Psychometric properties of the Social Phobia Inventory (SPIN). *Depression and Anxiety, 12*(2), 79–85. https://doi.org/10.1002/1520-6394(2000)12:2<79::AID-DA3>3.0.CO;2-6

Creamer, M., Bell, R., & Failla, S. (2003). Psychometric properties of the Impact of Event Scale–Revised. *Behaviour Research and Therapy, 41*(12), 1489–1496. https://doi.org/10.1016/j.brat.2003.07.010

Foa, E. B., Kozak, M. J., Salkovskis, P. M., Coles, M. E., & Amir, N. (1998). The validation of a new obsessive-compulsive disorder scale: The Obsessive-Compulsive Inventory. *Psychological Assessment, 10*(3), 206–214. https://doi.org/10.1037/1040-3590.10.3.206

Salkovskis, P. M., Rimes, K. A., Warwick, H. M. C., & Clark, D. M. (2002). The Health Anxiety Inventory: Development and validation of scales for the measurement of health anxiety and hypochondriasis. *Psychological Medicine, 32*(5), 843–853. https://doi.org/10.1017/S0033291702005822

Shear, M. K., Brown, T. A., Barlow, D. H., Money, R., Sholomskas, D. E., & Woods, S. W. (1997). Multicenter collaborative Panic Disorder Severity Scale. *The American Journal of Psychiatry, 154*(11), 1571–1575. https://doi.org/10.1176/ajp.154.11.1571

|  | Minimally adjusted model ^a^ | | | Fully adjusted Model ^b^ | | | Also adjusted for treatment-related factors ^c^ | | | Also adjusted for month and year of treatment ^d^ | | |
| --- | --- | --- | --- | --- | --- | --- | --- | --- | --- | --- | --- | --- |
|  | *N* | *Odds Ratio (95%CI)* | *p-value* | *N* | *Odds Ratio (95%CI)* | *p-value* | *N* | *Odds Ratio (95%CI)* | *p-value* | *N* | *Odds Ratio (95%CI)* | *p-value* |
| **Primary outcome** | | | | | | | | | | | | |
| ***Reliable recovery***  Gay men  Bisexual men | 29236 | 1.27 (1.17-1.38)  1.08 (0.91-1.29) | <0.001  0.361 | 29236 | 1.23 (1.13-1.34)  1.12 (0.94-1.33) | <0.001  0.217 | 29236 | 1.23 (1.13-1.34)  1.10 (0.92-1.31) | <0.001  0.318 | 29236 | 1.23 (1.13-1.33)  1.09 (0.91-1.34) | <0.001  0.351 |
| **Secondary outcomes** | | | | | | | | | | | | |
| ***Reliable Improvement***  Gay men  Bisexual men | 29299 | 1.21 (1.10-1.32)  1.07 (0.89-1.29) | <0.001  0.460 | 29299 | 1.16 (1.06-1.28)  1.07 (0.89-1.30) | 0.002  0.459 | 29299 | 1.16 (1.05-1.27)  1.04 (0.86-1.26) | 0.003  0.680 | 31171 | 1.16 (1.05, 1.27)  1.04 (0.85-1.26) | 0.003  0.720 |
| ***Attrition***  Gay men  Bisexual men | 26719 | 0.87 (0.79-0.96)  0.76 (0.61-0.94) | 0.007  0.010 | 26719 | 0.88 (0.80-0.97)  0.67 (0.54-0.83) | 0.013  <0.001 | 26719 | 0.88 (0.79-0.99)  0.70 (0.54-0.90) | 0.036  0.005 | 28347 | 0.88 (0.77-0.99)  0.71 (0.56-0.92) | 0.036  0.008 |
|  |  | Mean Difference  *(95%CI)* |  |  | Mean Difference *(95%CI)* |  |  | Mean Difference *(95%CI)* |  |  | Mean Difference *(95%CI)* |  |
| ***PHQ-9 Change***  Gay men  Bisexual men | 29290 | 0.67 (0.43-0.90)  -0.16 (-0.65-0.33) | <0.001  0.528 | 29290 | 0.51 (0.28-0.74)  -0.14 (-0.61-0.34) | <0.001  0.574 | 29290 | 0.49 (0.26-0.71)  -0.20 (-0.67-0.27) | <0.001  0.406 | 29290 | 0.48 (0.26-0.71)  -0.22 (-0.69-0.24) | <0.001  0.349 |
| ***GAD-7 Change***  Gay men  Bisexual men | 29288 | 0.58 (0.37-0.78)  0.15 (-0.28-0.58) | <0.001  0.483 | 29288 | 0.45 (0.25-0.65)  0.17 (-0.25-0.59) | <0.001  0.431 | 29288 | 0.43 (0.23-0.63)  0.12 (-0.30-0.53) | <0.001  0.582 | 29288 | 0.42 (0.23-0.62)  0.09 (-0.32-0.51) | <0.001  0.660 |
| ***Engagement***  Gay men  Bisexual men | 29285 | 0.01 (0.00-0.02)  0.02 (0.00-0.03) | 0.012  0.032 | 29285 | 0.01 (0.00-0.01)  0.02 (0.01-0.04) | 0.058  0.001 | 29285 | 0.06 (0.00-0.01)  0.02 (0.01-0.03) | 0.064  0.002 | 29285 | - 1. (0.00-0.01)   0.02 (0.01-0.03) | 0.080  0.007 |

### **Table 5.** Comparison of Clinical Outcomes for Gay and Bisexual Men Relative to Heterosexual Men.

Heterosexual men are the reference group for all comparisons. ^a^ Adjusted for pre-treatment scores (PHQ-9, GAD-7 or ADSM). ^b^ Additionally adjusted for age at referral, ethnicity, index of multiple deprivation (IMD) decile, being prescribed psychotropic medication alongside psychological therapy, long-term health condition status, personal functioning, problem descriptor and employment status. ^c^ Additionally adjusted for number of sessions and at which treatment intensity (high/low) and the number of weeks between referral to the services and the first treatment session. ^d^ Additionally adjusted for month and year of treatment.

### **Table 6.** Comparison of Clinical Outcomes for Lesbian and Bisexual Women Relative to Heterosexual Women.

|  | Minimally adjusted model ^a^ | | | Fully adjusted Model ^b^ | | | Also adjusted for treatment-related factors ^c^ | | | Also adjusted for month and year of treatment ^d^ | | |
| --- | --- | --- | --- | --- | --- | --- | --- | --- | --- | --- | --- | --- |
|  | *N* | *Odds Ratio (95%CI)* | *p-value* | *N* | *Odds Ratio (95%CI)* | *p-value* | *N* | *Odds Ratio (95%CI)* | *p-value* | *N* | *Odds Ratio (95%CI)* | *p-value* |
| **Primary outcome** | | | | | | | | | | | | |
| ***Reliable recovery***  Lesbian women  Bisexual women | 64841 | 1.11 (0.99-1.24)  0.98 (0.91-1.06) | 0.077  0.593 | 64841 | 1.09 (0.97-1.22)  0.99 (0.92-1.07) | 0.148  0.850 | 64841 | 1.07 (0.95-1.21)  0.96 (0.88-1.04) | 0.247  0.273 | 64841 | 1.07 (0.95-1.21)  0.93 (0.86-1.01) | 0.243  0.105 |
| **Secondary outcomes** | | | | | | | | | | | | |
| ***Reliable Improvement***  Lesbian women  Bisexual women | 64939 | 1.04 (0.92-1.18)  1.08 (0.99-1.17) | 0.541  0.077 | 64939 | 1.01 (0.89-1.14)  1.05 (0.96-1.15) | 0.925  0.264 | 64939 | 0.99 (0.87-1.12)  1.01 (0.92-1.10) | 0.824  0.890 | 64939 | 0.99 (0.87-1.12)  0.98 (0.89-1.07) | 0.825  0.614 |
| ***Attrition***  Lesbian women  Bisexual women | 59434 | 0.93 (0.82-1.07)  0.94 (0.86-1.03) | 0.302  0.202 | 59434 | 0.92 (0.80-1.05)  0.84 (0.77-0.93) | 0.217  <0.001 | 59434 | 0.98 (0.84-1.15)  0.94 (0.84-1.05) | 0.804  0.277 | 59434 | 0.98 (0.84-1.15)  0.97 (0.87-1.09) | 0.820  0.622 |
|  |  | *Mean Difference (95%CI)* |  |  | *Mean Difference (95%CI)* |  |  | *Mean Difference (95%CI)* |  |  | *Mean Difference (95%CI)* |  |
| ***PHQ-9 Change***  Lesbian women  Bisexual women | 64928 | 0.27 (-0.05-0.60)  -0.10 (-0.31- 0.12) | 0.102  0.387 | 64928 | 0.13 (-0.19-0.44)  -0.18 (-0.40-0.04) | 0.426  0.097 | 62928 | 0.07 (-0.23-0.38)  -0.29 (-0.50--0.08) | 0.647  0.008 | 64928 | 0.08 (-0.23-0.38)  -0.36 (-0.57- -0.15) | 0.631  0.001 |
| ***GAD-7 Change***  Lesbian women  Bisexual women | 64917 | 0.32 (0.03-0.61)  0.19 (-0.01-0.39) | 0.032  0.061 | 64917 | 0.20 (-0.08-0.49)  0.12 (-0.08-0.31) | 0.163  0.242 | 64917 | 0.15 (-0.13-0.43)  0.02 (-0.17-0.21) | 0.286  0.839 | 64917 | 0.15 (-0.13-0.43)  -0.05 (-0.24-0.15) | 0.285  0.628 |
| ***Engagement***  Lesbian women  Bisexual women | 64907 | 0.02 (0.01-0.03)  0.02 (0.01-0.03) | <0.001  <0.001 | 64907 | 0.02 (0.01-0.03)  0.03 (0.02-0.03) | <0.001  <0.001 | 64907 | 0.02 (0.01-0.02)  0.02 (0.01-0.03) | 0.001  <0.001 | 64907 | - 1. (0.01-0.02)   0.02 (0.01-0.02) | 0.001  <0.001 |

Heterosexual women are the reference group for all comparisons. ^a^ Adjusted for pre-treatment scores (PHQ-9, GAD-7 or ADSM). ^b^ Additionally adjusted for age at referral, ethnicity, index of multiple deprivation (IMD) decile, being prescribed psychotropic medication alongside psychological therapy, long-term health condition status, personal functioning, problem descriptor and employment status. ^c^ Additionally adjusted for number of sessions and at which treatment intensity (high/low) and the number of weeks between referral to the services and the first treatment session. ^d^ Additionally adjusted for month and year of treatment.

### **Table 7.** Comparison of descriptive statistics between male patients who disclosed their sexual orientation (Heterosexual, Lesbian and Bisexual) to those who reported “unsure” or declined to answer.

|  | Men who disclosed their sexual orientation N (%) or Mean (s.d.) | Men who reported “unsure”  N (%) or Mean (s.d.) | Responses recorded as declined to answer  N (%) or Mean (s.d.) |
| --- | --- | --- | --- |
| Total | N=29,299 | N=317 | N=1,555 |
| Socio-demographics |  |  |  |
| Age | 38.0 (13.5) | 38.4 (15.9) | 37.3 (13.8) |
| Ethnicity |  |  |  |
| White | 19,332 (66.0) | 173 (54.6) | 791 (50.9) |
| Mixed | 1,617 (5.5) | 26 (8.2) | 64 (4.1) |
| Asian | 3,927 (13.4) | 31 (9.8) | 155 (10.0) |
| Black | 2,589 (8.8) | 29 (9.1) | 91 (5.9) |
| Chinese | 208 (0.7) | 3 (0.9) | 14 (0.9) |
| Other | 1,181 (4.0) | 21 (6.6) | 70 (4.5) |
| Missing | 445 (1.5) | 34 (10.7) | 370 (23.8) |
| Employment Status |  |  |  |
| Employed | 17,577 (60.0) | 140 (44.2) | 823 (52.9) |
| Unemployed | 2,752 (9.4) | 59 (18.6) | 239 (15.4) |
| Student | 1,757 (6.0) | 33 (10.4) | 167 (10.7) |
| Long-term sick | 2,145 (7.3) | 26 (8.2) | 123 (7.9) |
| Homemaker | 183 (0.6) | 3 (0.9) | 8 (0.5) |
| Not seeking work | 2,090 (7.1) | 24 (7.6) | 51 (3.3) |
| Volunteer | 115 (0.4) | 2 (0.6) | 7 (0.5) |
| Retired | 1,206 (4.1) | 20 (6.3) | 53 (3.4) |
| Missing | 1,474 (5.0) | 10 (3.2) | 84 (5.4) |
| IMD Decile |  |  |  |
| 1 (Most deprived) | 2,047 (7.0) | 28 (8.8) | 134 (8.6) |
| 2 | 5,924 (20.2) | 72 (22.7) | 375 (24.1) |
| 3 | 4,729 (16.1) | 68 (21.5) | 321 (20.6) |
| 4 | 3,375 (11.5) | 47 (14.8) | 237 (15.2) |
| 5 | 2,477 (8.5) | 27 (8.5) | 152 (9.8) |
| 6 | 2,356 (8.0) | 23 (7.3) | 135 (8.7) |
| 7 | 1,498 (5.1) | 17 (5.4) | 50 (3.2) |
| 8 | 1,481 (5.1) | 18 (5.7) | 72 (4.6) |
| 9 | 560 (1.9) | 5 (1.6) | 22 (1.4) |
| 10 (Least deprived) | 201 (0.7) | 0 (0.0) | 13 (0.8) |
| Missing | 4,651 (15.9) | 12 (3.8) | 44 (2.8) |
| Clinical Characteristics | |  |  |
| First session PHQ-9 | 15.1 (5.7) | 15.5 (5.7) | 15.1 (5.6) |
| First session GAD-7 | 13.7 (4.5) | 13.8 (4.7) | 13.5 (4.5) |
| Psychotropic Medication |  |  |  |
| Prescribed Not taking | 1,644 (5.6) | 24 (7.6) | 170 (10.9) |
| Prescribed and taking | 9,920 (33.9) | 122 (38.5) | 566 (36.4) |
| Not prescribed | 16,108 (55.0) | 151 (47.6) | 745 (47.9) |
| Missing | 1,627 (5.6) | 20 (6.3) | 74 (4.8) |
| Long term condition status |  |  |  |
| No | 15,621 (53.3) | 93 (29.3) | 592 (38.1) |
| Yes | 7,686 (26.2) | 110 (34.7) | 385 (24.8) |
| Missing | 5,992 (20.5) | 114 (36.0) | 578 (37.2) |
| Personal functioning (sum of items 2-5 on the WSAS) | 15.6 (7.6) | 15.8 (7.4) | 15.4 (7.5) |
| Problem descriptor |  |  |  |
| Depression | 13,737 (46.9) | 151 (47.6) | 564 (36.3) |
| Mixed anxiety and depression | 1,354 (4.6) | 15 (4.7) | 46 (3.0) |
| GAD | 3,980 (13.6) | 41 (12.9) | 183 (11.8) |
| OCD | 651 (2.2) | 10 (3.2) | 36 (2.3) |
| PTSD | 1,033 (3.5) | 10 (3.2) | 32 (2.1) |
| Social Phobia | 1,350 (4.6) | 10 (3.2) | 77 (5.0) |
| Other phobia or panic disorder | 1,610 (5.5) | 19 (6.0) | 76 (4.9) |
| Anxiety disorder not otherwise specified | 554 (1.9) | 2 (0.6) | 7 (0.5) |
| Missing | 5,030 (17.2) | 59 (18.6) | 534 (34.3) |
| Weeks between referral and assessment | 3.0 (3.8) | 4.0 (4.2) | 3.5 (4.6) |
| Weeks between assessment and entering | 9.1 (8.7) | 9.1 (9.2) | 7.8 (8.2) |
| Treatment Related | |  |  |
| Number of attended sessions | 7.6 (4.8) | 7.9 (4.7) | 7.6 (4.9) |
| Main treatment intensity |  |  |  |
| Low intensity | 13,790 (47.1) | 107 (33.8) | 606 (39.0) |
| High intensity | 14,273 (48.7) | 196 (61.8) | 877 (56.4) |
| Missing | 1,236 (4.2) | 14 (4.4) | 72 (4.6) |
| End of treatment factors | |  |  |
| Reliable Recovery |  |  |  |
| No | 15,137 (51.7) | 187 (59.0) | 869 (55.9) |
| Yes | 14,099 (48.1) | 130 (41.0) | 686 (44.1) |
| Missing | 63 (0.2) | 0 (0.0) | 0 (0.0) |
| Reliable Improvement |  |  |  |
| No | 8,758 (29.9) | 109 (34.4) | 497 (32.0) |
| Yes | 20,541 (70.1) | 208 (65.6) | 1,058 (68.0) |
| Attrition |  |  |  |
| No | 18,960 (64.7) | 215 (67.8) | 978 (62.9) |
| Yes | 7,759 (26.5) | 72 (22.7) | 363 (23.3) |
| Missing | 2,580 (8.8) | 30 (9.5) | 214 (13.8) |
| Proportion of offered sessions attended | 0.823 (0.171) | 0.824 (0.171) | 0.811 (0.178) |
| PHQ-9 change | 6.0 (6.4) | 5.4 (6.5) | 5.4 (6.1) |
| GAD-7 change | 5.5 (5.7) | 5.0 (5.9) | 5.0 (5.6) |
| PHQ-9 end | 9.2 (6.7) | 10.1 (7.2) | 9.6 (6.6) |
| GAD-7 end | 8.2 (5.7) | 8.9 (5.9) | 8.5 (5.6) |

### **Table 8.** Comparison of descriptive statistics between women patients who disclosed their sexual orientation (Heterosexual, Lesbian and Bisexual) to those who reported “unsure” or declined to answer.

|  | Women who disclosed their sexual orientation N (%) or Mean (s.d.) | Women who reported “unsure”  N (%) or Mean (s.d.) | Responses recorded as declined to answer  N (%) or Mean (s.d.) |
| --- | --- | --- | --- |
| Total | N=64,939 | N=868 | N=3,420 |
| Socio-demographics | | | |
| Age | 36.6 (13.5) | 33.9 (13.9) | 36.9 (14.2) |
| Ethnicity |  |  |  |
| White | 40,891 (63.0) | 464 (53.5) | 1,634 (47.8) |
| Mixed | 4,428 (6.8) | 61 (7.0) | 192 (5.6) |
| Asian | 7,763 (12.0) | 90 (10.4) | 284 (8.3) |
| Black | 7,616 (11.7) | 86 (9.9) | 323 (9.4) |
| Chinese | 677 (1.0) | 24 (2.8) | 44 (1.3) |
| Other | 2,672 (4.1) | 66 (7.6) | 189 (5.5) |
| Missing | 892 (1.4) | 77 (8.9) | 754 (22.0) |
| Employment Status |  |  |  |
| Employed | 36,974 (56.9) | 411 (47.4) | 1,784 (52.2) |
| Unemployed | 4,885 (7.5) | 101 (11.6) | 398 (11.6) |
| Student | 5,308 (8.2) | 154 (17.7) | 413 (12.1) |
| Long-term sick | 4,606 (7.1) | 54 (6.2) | 192 (5.6) |
| Homemaker | 3,308 (5.1) | 32 (3.7) | 115 (3.4) |
| Not seeking work | 3,551 (5.5) | 50 (5.8) | 136 (4.0) |
| Volunteer | 311 (0.5) | 6 (0.7) | 13 (0.4) |
| Retired | 2,647 (4.1) | 26 (3.0) | 143 (4.2) |
| Missing | 3,349 (5.2) | 34 (3.9) | 226 (6.6) |
| IMD Decile |  |  |  |
| 1 (Most deprived) | 4,722 (7.3) | 89 (10.3) | 324 (9.5) |
| 2 | 13,150 (20.2) | 212 (24.4) | 784 (22.9) |
| 3 | 10,536 (16.2) | 178 (20.5) | 671 (19.6) |
| 4 | 7,652 (11.8) | 121 (13.9) | 488 (14.3) |
| 5 | 5,458 (8.4) | 75 (8.6) | 383 (11.2) |
| 6 | 4,852 (7.5) | 74 (8.5) | 280 (8.2) |
| 7 | 3,236 (5.0) | 49 (5.6) | 147 (4.3) |
| 8 | 3,143 (4.8) | 34 (3.9) | 150 (4.4) |
| 9 | 1,210 (1.9) | 9 (1.0) | 59 (1.7) |
| 10 (Least deprived) | 408 (0.6) | 3 (0.3) | 14 (0.4) |
| Missing | 10,572 (16.3) | 24 (2.8) | 120 (3.5) |
| First session PHQ-9 | 15.1 (5.6) | 15.5 (5.4) | 15.1 (5.5) |
| First session GAD-7 | 14.0 (4.4) | 13.8 (4.5) | 13.7 (4.5) |
| Psychotropic Medication |  |  |  |
| Prescribed Not taking | 3,769 (5.8) | 42 (4.8) | 292 (8.5) |
| Prescribed and taking | 20,037 (30.9) | 279 (32.1) | 1,120 (32.7) |
| Not prescribed | 36,874 (56.8) | 483 (55.6) | 1,754 (51.3) |
| Missing | 4,259 (6.6) | 64 (7.4) | 254 (7.4) |
| Long term condition status |  |  |  |
| No | 33,558 (51.7) | 267 (30.8) | 1,283 (37.5) |
| Yes | 16,976 (26.1) | 271 (31.2) | 851 (24.9) |
| Missing | 14,405 (22.2) | 330 (38.0) | 1,286 (37.6) |
| Personal functioning (sum of items 2-5 on the WSAS) | 15.5 (7.6) | 16.0 (7.6) | 15.6 (7.6) |
| Problem descriptor |  |  |  |
| Depression | 29,005 (44.7) | 361 (41.6) | 1,262 (36.9) |
| Mixed anxiety and depression | 3,284 (5.1) | 41 (4.7) | 112 (3.3) |
| GAD | 11,287 (17.4) | 135 (15.6) | 541 (15.8) |
| OCD | 1,313 (2.0) | 24 (2.8) | 60 (1.8) |
| PTSD | 2,173 (3.3) | 38 (4.4) | 98 (2.9) |
| Social Phobia | 1,733 (2.7) | 36 (4.1) | 111 (3.2) |
| Other phobia or panic disorder | 3,543 (5.5) | 47 (5.4) | 136 (4.0) |
| Anxiety disorder not otherwise specified | 1,080 (1.7) | 1 (0.1) | 17 (0.5) |
| Missing | 11,521 (17.7) | 185 (21.3) | 1,083 (31.7) |
| Weeks between referral and assessment | 3.2 (3.9) | 4.6 (5.8) | 3.7 (4.2) |
| Weeks between assessment and entering | 9.3 (9.0) | 10.2 (10.2) | 8.1 (8.5) |
| Treatment Related | |  |  |
| Number of attended sessions | 7.9 (4.9) | 8.2 (4.9) | 7.7 (4.9) |
| Main treatment intensity |  |  |  |
| Low intensity | 29,349 (45.2) | 284 (32.7) | 1,333 (39.0) |
| High intensity | 32,970 (50.8) | 544 (62.7) | 1,980 (57.9) |
| Missing | 2,620 (4.0) | 40 (4.6) | 107 (3.1) |
| End of treatment factors | |  |  |
| Reliable Recovery |  |  |  |
| No | 34,429 (53.0) | 527 (60.7) | 1,983 (58.0) |
| Yes | 30,412 (46.8) | 340 (39.2) | 1,431 (41.8) |
| Missing | 98 (0.2) | 1 (0.1) | 6 (0.2) |
| Reliable Improvement |  |  |  |
| No | 19,289 (29.7) | 308 (35.5) | 1,167 (34.1) |
| Yes | 45,650 (70.3) | 560 (64.5) | 2,253 (65.9) |
| Attrition |  |  |  |
| No | 42,080 (64.8) | 581 (66.9) | 2,223 (65.0) |
| Yes | 17,354 (26.7) | 200 (23.0) | 823 (24.1) |
| Missing | 5,505 (8.5) | 87 (10.0) | 374 (10.9) |
| Proportion of offered sessions attended | 0.813 (0.172) | 0.820 (0.168) | 0.814 (0.176) |
| PHQ-9 change | 5.8 (6.3) | 5.2 (6.0) | 5.2 (6.3) |
| GAD-7 change | 5.6 (5.8) | 4.8 (5.6) | 4.9 (5.8) |
| PHQ-9 end | 9.3 (6.5) | 10.2 (6.6) | 10.0 (6.6) |
| GAD-7 end | 8.5 (5.8) | 9.0 (5.7) | 8.9 (5.8) |

|  | Minimally adjusted model ^a^ | | | Fully adjusted Model ^b^ | | | Also adjusted for treatment-related factors ^c^ | | | Also adjusted for month and year of treatment ^d^ | | |
| --- | --- | --- | --- | --- | --- | --- | --- | --- | --- | --- | --- | --- |
|  | *N* | *OR or Mean Difference (95%CI)* | *p-value* | *N* | *OR or Mean Difference (95%CI)* | *p-value* | *N* | *OR or Mean Difference (95%CI)* | *p-value* | *N* | *OR or Mean Difference (95%CI)* | *p-value* |
| **Primary outcome** | | | | | | | | | | | | |
| ***Reliable recovery***  Unsure  Declined to answer | 31108 | 0.81 (0.64 - 1.02)  0.90 (0.81 - 1.01) | 0.075  0.063 | 31108 | 0.89 (0.70 - 1.13)  0.95 (0.85 - 1.06) | 0.326  0.373 | 31108 | 0.88 (0.70 - 1.12)  0.96 (0.86 - 1.07) | 0.308  0.480 | 31108 | 0.88 (0.69-1.12)  0.98 (0.87-1.09) | 0.299  0.676 |
| **Secondary outcomes** | | | | | | | | | | | | |
| ***Reliable Improvement***  Unsure  Declined to answer | 31171 | 0.83 (0.66 - 1.06)  0.94 (0.84 - 1.05) | 0.130  0.267 | 31171 | 0.92 (0.73 - 1.17)  0.99 (0.88 - 1.11) | 0.511  0.812 | 31171 | 0.92 (0.72 - 1.17)  1.01 (0.90 - 1.13) | 0.485  0.906 | 31108 | 0.91 (0.72-1.17)  1.02 (0.91-1.15) | 0.475  0.684 |
| ***PHQ-9 Change***  Unsure  Declined to answer | 31162 | -0.64 (-1.29 - 0.00)  -0.41 (-0.71- -0.10) | 0.051  0.009 | 31162 | -0.26 (-0.89 - 0.36)  -0.23 (-0.53 - 0.06) | 0.410  0.120 | 31162 | -0.28 (-0.90 - 0.33)  -0.21 (-0.50 - 0.08) | 0.365  0.153 | 31162 | -0.31 (-0.92-0.31)  -0.17 (-0.46-0.12) | 0.329  0.251 |
| ***GAD-7 Change***  Unsure  Declined to answer | 31159 | -0.50 (-1.06 - 0.07)  -0.32 (-0.59 - -0.06) | 0.085  0.017 | 31159 | -0.21 (-0.76 - 0.35)  -0.18 (-0.44 - 0.08) | 0.463  0.165 | 31159 | -0.22 (-0.77 - 0.32)  -0.16 (-0.42 - 0.09) | 0.422  0.209 | 31159 | -0.25 (-0.79- 0.29)  -0.12 (-0.37-0.14) | 0.364  0.365 |
| ***Engagement***  Unsure  Declined to answer | 31157 | 0.01 (-0.01 - 0.03)  0.00 (-0.01 - 0.01) | 0.284  0.531 | 31157 | 0.01 (-0.01 - 0.03)  0.00 (-0.01 - 0.01) | 0.193  0.837 | 31157 | 0.01 (-0.01 - 0.03)  0.00 (-0.01 - 0.01) | 0.220  0.878 | 31157 | - 1. (-0.01-0.03)   0.00 (-0.01-0.01) | 0.340  0.663 |
| ***Attrition***  Unsure  Declined to answer | 28347 | 0.90 (0.68 - 1.18)  1.05 (0.93 - 1.19) | 0.431  0.431 | 28347 | 0.84 (0.64 - 1.11)  1.03 (0.90 - 1.17) | 0.222  0.683 | 28347 | 0.87 (0.63 - 1.19)  0.98 (0.85 - 1.14) | 0.374  0.819 | 28347 | 0.89 (0.65-1.22)  0.98 (0.84-1.13) | 0.468  0.752 |

### **Table 9.** Sensitivity Analysis that Compares Clinical Outcomes Between Men Who Reported "Unsure" or "Declined to Answer" and Those Who Disclosed Their Sexual Orientation (Heterosexual, Gay, and Bisexual).

Heterosexual, Gay, and Bisexual men are the reference group for all comparisons. ^a^ Adjusted for pre-treatment scores (PHQ-9, GAD-7 or ADSM). ^b^ Additionally adjusted for age at referral, ethnicity, index of multiple deprivation (IMD) decile, being prescribed psychotropic medication alongside psychological therapy, long-term health condition status, personal functioning, problem descriptor and employment status. ^c^ Additionally adjusted for number of sessions and at which treatment intensity (high/low) and the number of weeks between referral to the services and the first treatment session. ^d^ Additionally adjusted for month and year of treatment.

### **Table 10.** Sensitivity Analysis that Compares Clinical Outcomes Between Women Who Reported "Unsure" or "Declined to Answer" and Those Who Disclosed Their Sexual Orientation (Heterosexual, Gay, and Bisexual).

|  | Minimally adjusted model ^a^ | | | Fully adjusted Model ^b^ | | | Also adjusted for treatment-related factors ^c^ | | | Also adjusted for month and year of treatment ^d^ | | |
| --- | --- | --- | --- | --- | --- | --- | --- | --- | --- | --- | --- | --- |
|  | *N* | *OR or Mean Difference (95%CI)* | *p-value* | *N* | *OR or Mean Difference (95%CI)* | *p-value* | *N* | *OR or Mean Difference (95%CI)* | *p-value* | *N* | *OR or Mean Difference (95%CI)* | *p-value* |
| **Primary outcome** | | | | | | | | | | | | |
| ***Reliable recovery***  Unsure  Declined to answer | 69122 | 0.80 (0.70 - 0.92)  0.88 (0.82 - 0.95) | 0.002  0.001 | 69122 | 0.87 (0.75 - 1.00)  0.91 (0.84 - 0.98) | 0.053  0.009 | 69122 | 0.86 (0.74 - 0.99)  0.92 (0.85 - 0.99) | 0.037  0.025 | 69122 | 0.84 (0.73- 0.98)  0.93 (0.87- 1.01) | 0.023  0.072 |
| **Secondary Outcomes** | | | | | | | | | | | | |
| ***Reliable Improvement***  Unsure  Declined to answer | 69227 | 0.81 (0.70 - 0.93)  0.85 (0.79 - 0.92) | 0.003  <0.001 | 69227 | 0.86 (0.74 - 0.99)  0.88 (0.81 - 0.94) | 0.035  0.001 | 69227 | 0.84 (0.72 - 0.97)  0.89 (0.82 - 0.96) | 0.020  0.003 | 69227 | 0.83 (0.71-0.96)  0.91 (0.84-0.98) | 0.011  0.013 |
| ***PHQ-9 Change***  Unsure  Declined to answer | 69216 | -0.65 (-1.04 - 0.26)  -0.57 (-0.77 - 0.37) | 0.001  <0.001 | 69216 | -0.40 (-0.77 - -0.02)  -0.45 (-0.65 - -0.26) | 0.039  <0.001 | 69216 | -0.42 (-0.79 - -0.05)  -0.42 (-0.62 - -0.23) | 0.027  <0.001 | 69216 | -0.46 (-0.83- -0.09)  -0.37 (-0.56 –0.18) | 0.014  <0.001 |
| ***GAD-7 Change***  Unsure  Declined to answer | 69203 | -0.36 (-0.72 - 0.01)  -0.35 (-0.54 - 0.17) | 0.043  <0.001 | 69203 | -0.17 (-0.51 - 0.17)  -0.28 (-0.46 - -0.10) | 0.333  0.002 | 69203 | -0.19 (-0.53 - 0.15)  -0.24 (-0.42 - -0.07) | 0.276  0.007 | 69203 | -0.23 (-0.57- 0.10)  -0.20 (-0.37- -0.02) | 0.173  0.029 |
| ***Engagement***  Unsure  Declined to answer | 69195 | 0.01 (0.00 - 0.2)  0.01 (0.00 - 0.01) | 0.034  0.034 | 69195 | 0.02 (0.00 - 0.03)  0.01 (0.00 - 0.01) | 0.006  0.019 | 69195 | 0.01 (0.00 - 0.03)  0.01 (0.00 - 0.01) | 0.007  0.006 | 69195 | - 1. (0.00-0.02)   0.01 (0.00, 0.02) | 0.032  <0.001 |
| ***Attrition***  Unsure  Declined to answer | 63261 | 0.04 (0.79 - 1.10)  1.03 (0.95 - 1.12) | 0.424  0.483 | 63261 | 0.89 (0.75 - 1.05)  1.13 (0.94 - 1.12) | 0.169  0.554 | 63261 | 0.95 (0.78 - 1.16)  1.00 (0.90 - 1.10) | 0.608  0.942 | 63261 | 0.97 (0.80-1.18)  0.98 (0.89-1.08) | 0.788  0.716 |

Heterosexual, lesbian and bisexual women are the reference group for all comparisons. ^a^ Adjusted for pre-treatment scores (PHQ-9, GAD-7 or ADSM). ^b^ Additionally adjusted for age at referral, ethnicity, index of multiple deprivation (IMD) decile, being prescribed psychotropic medication alongside psychological therapy, long-term health condition status, personal functioning, problem descriptor and employment status. ^c^ Additionally adjusted for number of sessions and at which treatment intensity (high/low) and the number of weeks between referral to the services and the first treatment session. ^d^ Additionally adjusted for month and year of treatment.

### **Table 11.** Comparison of Clinical Outcomes for Gay and Bisexual Men Relative to Heterosexual Men. (Complete Case Analysis)

|  | Minimally adjusted model ^a^ | | | Fully adjusted Model ^b^ | | | Also adjusted for treatment-related factors ^c^ | | | Also adjusted for month and year of treatment ^d^ | | |
| --- | --- | --- | --- | --- | --- | --- | --- | --- | --- | --- | --- | --- |
|  | *N* | *OR or Mean Difference (95%CI)* | *p-value* | *N* | *OR or Mean Difference (95%CI)* | *p-value* | *N* | *OR or Mean Difference (95%CI)* | *p-value* | *N* | *OR or Mean Difference (95%CI)* | *p-value* |
| **Primary outcome** | | | | | | | | | | | | |
| ***Reliable recovery***  Gay men  Bisexual men | 29,224 | 1.27 (1.16-1.38)  1.08 (0.91-1.29) | <0.001  0.361 | 14,978 | 1.24 (1.08-1.42)  0.96 (0.72-1.28) | 0.002  0.802 | 13,337 | 1.24 (1.07-1.44)  1.00 (0.74-1.35) | 0.004  0.992 | 13,337 | 1.24 (1.07-1.44)  0.99 (0.73-1.35) | 0.004  0.966 |
| **Secondary outcomes** | | | | | | | | | | | | |
| ***Reliable Improvement***  Gay men  Bisexual men | 29,287 | 1.21 (1.10-1.33)  1.07 (0.89-1.30) | <0.001  0.454 | 15,007 | 1.30 (1.11-1.52)  1.01 (0.75-1.38) | 0.001  0.932 | 13,366 | 1.36 (1.14-1.61)  1.09 (0.77-1.53) | <0.001  0.634 | 13,366 | 1.35 (1.14-1.61)  1.08 (0.77-1.53) | 0.001  0.646 |
| ***PHQ-9 Change***  Gay men  Bisexual men | 29,287 | 0.67 (0.43-0.90)  -0.16 (-0.65-0.33) | <0.001  0.526 | 15,007 | 0.51 (0.13-0.88)  -0.49 (-1.28-0.31) | 0.008  0.234 | 13,366 | 0.49 (0.11-0.88)  -0.23 (-1.05-0.59) | 0.013  0.580 | 13,366 | 0.49 (0.11-0.88)  -0.25 (-1.07-0.57) | 0.012  0.553 |
| ***GAD-7 Change***  Gay men  Bisexual men | 29,287 | 0.58 (0.37-0.78)  0.15 (-0.28-0.58) | <0.001  0.484 | 15,007 | 0.47 (0.14-0.80)  -0.08 (-0.79-0.62) | 0.005  0.816 | 13,366 | 0.47(0.13-0.81)  0.07 (-0.65-0.79) | 0.007  0.856 | 13,366 | 0.47 (0.13-0.81)  0.05 (-0.67-0.77) | 0.007  0.894 |
| ***Engagement***  Gay men  Bisexual men | 29,273 | - 1. (0.00-0.02)   0.02 (0.00-0.03) | 0.013  0.033 | 15,007 | - 1. (-0.01-0.02)   0.02 (0.00-0.04) | 0.325  0.076 | 13,366 | 0.00 (-0.01-0.01)  0.02 (0.00-0.05) | 0.716  0.034 | 13,366 | 0.00 (-0.01-0.01)  0.02 (0.00-0.04) | 0.723  0.052 |
| ***Attrition***  Gay men  Bisexual men | 26,707 | 0.87 (0.79 – 0.96)  0.75 (0.61 – 0.94) | 0.007  0.010 | 13,629 | 0.86 (0.73-1.01)  0.84 (0.60-1.16) | 0.061  0.288 | 12,361 | 0.87 (0.71-1.05)  0.87 (0.58-1.30) | 0.153  0.498 | 12,361 | 0.86 (0.71-1.05)  0.86 (0.58-1.29) | 0.139  0.477 |

Heterosexual men are the reference group for all comparisons. ^a^ Adjusted for pre-treatment scores (PHQ-9, GAD-7 or ADSM). ^b^ Additionally adjusted for age at referral, ethnicity, index of multiple deprivation (IMD) decile, being prescribed psychotropic medication alongside psychological therapy, long-term health condition status, personal functioning, problem descriptor and employment status. ^c^ Additionally adjusted for number of sessions and at which treatment intensity (high/low) and the number of weeks between referral to the services and the first treatment session. ^d^ Additionally adjusted for month and year of treatment.

### **Table 12.** Comparison of Clinical Outcomes for Lesbian and Bisexual Women Relative to Heterosexual Women (Complete Case Analysis)

|  | Minimally adjusted model ^a^ | | | Fully adjusted Model ^b^ | | | Also adjusted for treatment-related factors ^c^ | | | Also adjusted for month and year of treatment ^d^ | | |
| --- | --- | --- | --- | --- | --- | --- | --- | --- | --- | --- | --- | --- |
|  | *N* | *OR or Mean Difference (95%CI)* | *p-value* | *N* | *OR or Mean Difference (95%CI)* | *p-value* | *N* | *OR or Mean Difference (95%CI)* | *p-value* | *N* | *OR or Mean Difference (95%CI)* | *p-value* |
| **Primary outcome** | | | | | | | | | | | | |
| ***Reliable recovery***  Lesbian women  Bisexual women | 64,817 | 1.11 (0.99-1.24)  0.98 (0.91-1.06) | 0.077  0.590 | 32,263 | 1.12 (0.93-1.33)  0.93 (0.81-1.06) | 0.223  0.290 | 28,882 | 1.08 (0.90-1.31)  0.91 (0.79-1.05) | 0.405  0.192 | 28,882 | 1.09 (0.90-1.31)  0.90 (0.77-1.04) | 0.393  0.137 |
| **Secondary outcomes** | | | | | | | | | | | | |
| ***Reliable Improvement***  Lesbian women  Bisexual women | 64,915 | 1.04 (0.92-1.18)  1.08 (0.99-1.17) | 0.534  0.073 | 32,295 | 1.15 (0.94-1.40)  1.06 (0.91-1.23) | 0.167  0.438 | 28,911 | 1.13 (0.91-1.40)  1.05 (0.90-1.24) | 0.267  0.519 | 28,911 | 1.13 (0.91-1.40)  1.04 (0.88-1.22) | 0.267  0.668 |
| ***PHQ-9 Change***  Lesbian women  Bisexual women | 64,915 | 0.27 (-0.05-0.59)  -0.10 (-0.32-0.12) | 0.103  0.386 | 32,295 | 0.40 (-0.09-0.89)  -0.21 (-0.59-0.17) | 0.113  0.270 | 28,911 | 0.36 (-0.15-0.86)  -0.27 (-0.65-0.12) | 0.165  0.178 | 28,911 | 0.35 (-0.15-0.85)  -0.32 (-0.71-0.07) | 0.170  0.107 |
| ***GAD-7 Change***  Lesbian women  Bisexual women | 64,915 | 0.32 (0.03-0.61)  0.19 (-0.01-0.39) | 0.033  0.061 | 32,295 | 0.40 (-0.05-0.85)  0.05 (-0.29-0.39) | 0.079  0.779 | 28,911 | 0.27 (-0.18-0.73)  0.02 (-0.33-0.38) | 0.237  0.892 | 28,911 | 0.27 (-0.18-0.73)  -0.03 (-0.38-0.32) | 0.236  0.881 |
| ***Engagement***  Lesbian women  Bisexual women | 64,883 | 0.02(0.01-0.03)  0.02 (0.01-0.03) | <0.001  <0.001 | 32,295 | 0.02 (0.01-0.04)  0.03 (0.02-0.04) | 0.003  <0.001 | 28,911 | - 1. (0.00-0.03)   2. (0.01-0.03) | 0.036  <0.001 | 28,911 | - 1. (0.00-0.03)   2. (0.01-0.03) | 0.035  <0.001 |
| ***Attrition***  Lesbian women  Bisexual women | 59,412 | 0.93 (0.82-1.07)  0.94 (0.86-1.03) | 0.300  0.199 | 29,504 | 0.83 (0.67-1.01)  0.87 (0.74-1.01) | 0.068  0.075 | 26,841 | 0.94 (0.73-1.21)  1.00 (0.82-1.21) | 0.626  0.962 | 26,841 | 0.93 (0.72-1.21)  1.01 (0.83-1.23) | 0.597  0.892 |

Heterosexual women are the reference group for all comparisons. ^a^ Adjusted for pre-treatment scores (PHQ-9, GAD-7 or ADSM). ^b^ Additionally adjusted for age at referral, ethnicity, index of multiple deprivation (IMD) decile, being prescribed psychotropic medication alongside psychological therapy, long-term health condition status, personal functioning, problem descriptor and employment status. ^c^ Additionally adjusted for number of sessions and at which treatment intensity (high/low) and the number of weeks between referral to the services and the first treatment session. ^d^ Additionally adjusted for month and year of treatment.

**Table 13.** Exploratory Descriptives of Early Disengagement Before Treatment

|  | **Among Males** | **Among Females** |
| --- | --- | --- |
| **Proportion not attending NHS TTad following referral*** | **N (%)** | **N (%)** |
| Heterosexual | 28,532 (30.7) | 64,299 (30.3) |
| Gay/Lesbian | 3,111 (31.6) | 1,599 (33.1) |
| Bi-sexual | 888 (36.5) | 4,143 (36.2) |
| Not sure | 854 (47.1) | 2,097 (45.2) |
| Declined to answer | 3,760 (47.6) | 6,720 (42.7) |
| **Proportion attending only one session**** |  |  |
| Heterosexual | 29,799 (46.4) | 66,703 (45.1) |
| Gay/Lesbian | 3,203 (47.4) | 1,564 (48.4) |
| Bi-sexual | 827 (53.5) | 3,616 (49.6) |
| Not sure | 517 (53.8) | 1,328 (52.2) |
| Declined to answer | 1,993 (48.1) | 4,212 (46.7) |

Note: These figures were estimated before standard NHS TTad exclusions, such as excluding individuals who did not meet the clinical threshold for depression or anxiety at referral and referrals with presenting complaints for which there is no evidence-based psychological therapy offered in NHS TTad.

*Calculated among referrals not still in treatment (i.e. discharged from the services)

**Calculated among referrals attending at least one session and discharged
